# Nonlinear changes in delayed functional network topology in Alzheimer’s disease: relationship with amyloid and tau pathology

**DOI:** 10.1186/s13195-023-01252-3

**Published:** 2023-06-16

**Authors:** Mite Mijalkov, Dániel Veréb, Anna Canal-Garcia, Thomas Hinault, Giovanni Volpe, Joana B. Pereira, for the Alzheimer’s Disease Neuroimaging Initiative

**Affiliations:** 1 Department of Clinical Neuroscience, Karolinska Institutet, Stockholm, Sweden; 2 Normandie Univ, Unicaen, PSL, Université Paris, EPHE, Inserm, U1077, CHU de Caen, Centre Cyceron, 14000 Caen, France; 3 Department of Physics, Goteborg University, Goteborg, Sweden

**Keywords:** Anti-symmetric correlations, Delayed connectivity, Directed connectivity, Functional MRI, Functional integration, Nonlinear functional connectivity, Functional segregation, Network analysis, Alzheimer’s disease

## Abstract

**Background:**

Alzheimer’s disease is a neurodegenerative disorder associated with the abnormal deposition of pathological processes, such as amyloid-ß and tau, which produces nonlinear changes in the functional connectivity patterns between different brain regions across the Alzheimer’s disease continuum. However, the mechanisms underlying these nonlinear changes remain largely unknown. Here, we address this question using a novel method based on temporal or delayed correlations and calculate new whole-brain functional networks to tackle these mechanisms.

**Methods:**

To assess our method, we evaluated 166 individuals from the ADNI database, including amyloid-beta negative and positive cognitively normal subjects, patients with mild cognitive impairment, and patients with Alzheimer’s disease dementia. We used the clustering coefficient and the global efficiency to measure the functional network topology and assessed their relationship with amyloid and tau pathology measured by positron emission tomography, as well as cognitive performance using tests measuring memory, executive function, attention, and global cognition.

**Results:**

Our study found nonlinear changes in the global efficiency, but not in the clustering coefficient, showing that the nonlinear changes in functional connectivity are due to an altered ability of brain regions to communicate with each other through direct paths. These changes in global efficiency were most prominent in early disease stages. However, later stages of Alzheimer’s disease were associated with widespread network disruptions characterized by changes in both network measures. The temporal delays required for the detection of these changes varied across the Alzheimer’s disease continuum, with shorter delays necessary to detect changes in early stages and longer delays necessary to detect changes in late stages. Both global efficiency and clustering coefficient showed quadratic associations with pathological amyloid and tau burden as well as cognitive decline.

**Conclusions:**

This study suggests that global efficiency is a more sensitive indicator of network changes in Alzheimer’s disease when compared to clustering coefficient. Both network properties were associated with pathology and cognitive performance, demonstrating their relevance in clinical settings. Our findings provide an insight into the mechanisms underlying nonlinear changes in functional network organization in Alzheimer’s disease, suggesting that it is the lack of direct connections that drives these functional changes.

**Supplementary Information:**

The online version contains supplementary material available at 10.1186/s13195-023-01252-3.

## Background

While the exact mechanisms that determine the development of Alzheimer’s disease (AD) remain under debate, there is consistent evidence showing that the abnormal accumulation of amyloid-beta (Aß) and tau plays a crucial role [1]. This led to the development of several Aß and tau biomarkers to track the progression of AD, which have shown that Aβ deposition is one of the earliest events, followed by tau deposition [1–3]. These abnormalities have downstream effects on several physiological processes, including the communication between brain regions or functional connections [4, 5].

In particular, emerging evidence suggests that early Aβ deposition is associated with increased brain activity in cognitively normal individuals with Aβ burden [6–8] as well as in individuals at the earliest stages of mild cognitive impairment (MCI) [9–11]. This increased brain activity has been interpreted as a reflection of maladaptive, excitotoxic processes preceding neuronal loss, as a compensatory mechanism in response to the adverse effects of Aβ buildup on neuronal function, or as a protraction of increases in activation occurring in cognitively healthy individuals [12–14]. As the disease progresses, these increases are followed by a decrease in brain activation, together with cognitive decline [11, 14, 15]. This loss of connectivity has been related to the progressive loss of structure and neuronal death induced by tau pathology, which destabilizes axonal structure and disrupts axonal transport [5, 16]. Together these findings indicate a nonlinear trajectory of functional changes throughout the AD spectrum, with increases of neuronal hyperexcitability being followed by hypoconnectivity or loss of functional connections.

Although a few studies have assessed these functional nonlinear changes in connectivity on functional magnetic resonance imaging (fMRI) [5, 13], the mechanisms underlying this nonlinearity remain largely unknown. Specifically, it is unclear whether these changes are due to alterations in the clusters of connections between neighboring areas or due to changes in the communication between distant brain regions. Moreover, previous studies assessing cross-sectional functional nonlinearity in AD are limited by the fact that they evaluated connectivity as a static phenomenon that does not change over the course of the functional MRI scan [17, 18]. This contrasts with emerging evidence showing that functional connectivity is a dynamic process that can be characterized by time delays between the activation of different brain regions [17, 19–21]. These time delays can be regarded as a measure of the “functional distance” between such regions, with those that are closely connected becoming co-activated after a shorter delay, whereas those that are more distantly connected get activated after longer delays [19, 21, 22]. Capturing this information from functional connectivity is crucial for a deeper understanding of brain function, as changes in temporal dynamics have been proposed to critically underly brain changes associated with healthy aging and dementia [23].

Here, we employed temporal delays to assess the strength and direction of the pairwise functional connectivity between all brain regions. To quantify this delayed functional connectivity, we evaluated the correlation between the activity in one region and the subsequent, or delayed, activity in the other region. We considered multiple temporal delays, with each delay corresponding to a 3-s interval. Our study focused on measuring the changes in the network organization of such delayed functional networks across the AD continuum, including cognitively normal subjects, patients with MCI, and patients with AD dementia, as well as their association with the temporal delay at which the networks were calculated. In addition, we evaluated the relationship between time-varying functional network measures with amyloid and tau pathology measured on positron emission tomography as well as cognitive functions. Network organization was assessed using two measures, the clustering coefficient and global efficiency. The clustering coefficient reflects the extent to which neighboring brain areas form clusters of connections, promoting specialization. In contrast, the global efficiency reflects whether brain regions are directly connected between them or indirectly connected, promoting integration. Healthy brain networks exhibit both high clustering and global efficiency, facilitating optimal segregation and integration, which are crucial for normal brain functioning [24]. These measures are the most representative measures of network segregation and integration and among the most commonly used measures in the literature, which allowed us to understand better our results in the context of previous studies [18, 25].

Using these methods, this study aimed to explore the mechanisms underlying the nonlinear changes in functional network organization observed throughout the AD continuum. Specifically, we investigated whether these changes primarily stemmed from alterations in communication between distant brain areas (global efficiency) or neighboring brain areas (clustering coefficient). We hypothesized that both measures will show distinct trajectories across the AD continuum that are influenced by the temporal delay used to calculate functional connectivity. Furthermore, we expected that network measures will show significant associations with elevated levels of Aβ and tau pathology, as well as cognitive decline, indicating their relevance in clinical settings as biomarkers to track and predict disease severity.

## Methods

### Participants

We included 166 participants from the Alzheimer’s Disease Neuroimaging Initiative 3 (ADNI3) cohort. All participants underwent functional MRI, amyloid-PET (^18^F-Florbetapir), and tau-PET (18F-Flortaucipir) scans, which were performed within 1 year of the functional MRI scan for the majority of the participants. The inclusion/exclusion criteria for ADNI can be found at: http://www.adni-info.org/. In summary, participants had to be fluent in Spanish or English, be aged between 55 and 90 years, have completed a minimum of 6 years of education, and do not present significant neurological disorders other than AD. The controls were included if they scored between 24 and 30 on the Mini-Mental State Examination (MMSE) and had a Clinical Dementia Rating-Sum of Boxes (CDR-SB) score of 0, with no depression, MCI, or dementia. MCI participants were selected based on the Peterson criteria [26] for amnestic MCI. AD participants met the National Institute for Neurological and Communicative Disorders and Stroke-Alzheimer’s Disease and Related Disorder Association (NINDS/ADRDA) criteria for probable AD, scored between 18 and 26 on the MMSE and had a CDR-SB score ranging from 0.5 to 1.0. We only included participants with scores on the category fluency animal naming (CF), trail making test parts A and B (Trail A and Trail B), 13-item Alzheimer’s Disease Assessment Scale–Cognitive Subscale (ADAS-Cog 13), delayed word recall (ADAS Q4), and the Modified Preclinical Alzheimer Cognitive Composite with Trails test (mPACCtrailsB) since these tests are the most commonly used in AD clinical trials [27, 28].

ADNI was launched in 2003 as a public–private partnership, led by Principal Investigator Michael W. Weiner, MD. The primary goal of ADNI has been to test whether serial MRI, PET, other biological markers, and clinical and neuropsychological assessment can be combined to measure the progression of MCI and early AD. ADNI is conducted in accordance with the ethical standards of the institutional research committees and with the 1975 Helsinki Declaration and its later amendments. Written informed consent was obtained from all subjects and/or authorized representatives and study partners. Ethical permits have been obtained at each participating site of ADNI and we have signed the data user agreements to analyze the data.

### Group classification

We classified the participants based on clinical diagnosis and Aβ-PET levels using a cut-off > 1.11 based on earlier research showing that Aβ deposition is one of the earliest events in AD [3]. This resulted (Table 1) in 81 Aβ-negative cognitively normal (CN Aβ −) individuals, 36 Aβ-positive cognitively normal (CN Aβ +) individuals, 31 Aβ-positive patients with mild cognitive impairment (MCI Aβ +), and 18 Aβ-positive patients with AD dementia (AD Aβ +). Individuals with MCI and AD without Aβ pathology were excluded since they are not considered to be part of the AD continuum and may have a non-AD disorder [1].

**Table 1:** Characteristics of the sample

	**CN Aβ − (*n* = 81)**	**CN Aβ + (*n* = 36)**	**MCI Aβ + (*n* = 31)**	**AD Aβ + (*n* = 18)**	***p*-values**
Age	75.5 (32.5)	78.0 (27.8)	79.5 (30.1)	84.5 (29.3)	< 0.001
Sex (M/F)	34/47	19/17	16/15	9/9	0.654
Education	18.0 (10.0)	16.0 (8.0)	16.0 (12.0)	15.0 (7.0)	0.017
APOE ε4	63/17/1	19/16/1	14/10/7	11/6/1	< 0.001
Global Aβ SUVR	1.0 (0.2)	1.3 (0.6)	1.4 (1.2)	1.4 (0.8)	< 0.001
Braak I–II SUVR	1.1 (0.5)	1.1 (0.6)	1.3 (1.0)	1.5 (1.9)	< 0.001
Braak III–IV SUVR	1.1 (0.5)	1.2 (0.4)	1.2 (0.9)	1.4 (2.0)	< 0.001
Braak V–VI SUVR	1.0 (0.4)	1.0 (0.4)	1.1 (0.9)	1.1 (1.2)	< 0.001
ADAS 13	12.0 (21.7)	13.3 (27.3)	19.3 (27.0)	32.3 (26.7)	< 0.001
ADAS Q4	2.0 (6.0)	3.0 (7.0)	5.0 (7.0)	8.0 (5.0)	< 0.001
mPACC	1.5 (15.5)	− 0.4 (19.0)	− 3.9 (16. 9)	− 14.9 (26.4)	< 0.001
CF	22 (28)	22 (22)	16 (20)	13.5 (14)	< 0.001
Trail A	30 (48)	32 (37)	39 (50)	51.5 (129)	< 0.001
Trail B	2 (6)	3 (7)	5 (7)	8 (5)	< 0.001

Medians for each group are followed by range of values in parenthesis, except for sex. APOE ε4 values show the number of ε4 copies (zero/one/two). Comparisons between groups were performed using Kruskal–Wallis tests for continuous variables and with chi-squared tests for binary variables. CN, cognitively normal; MCI, mild cognitive impairment; AD, Alzheimer’s disease; Aβ, amyloid-β; APOE ε4, apolipoprotein E ε4; ADAS 13, 13-item Alzheimer’s Disease Assessment Scale–Cognitive Subscale; ADAS Q4, delayed word recall item of the Alzheimer’s Disease Assessment Scale–Cognitive Subscale; mPACC, Modified Preclinical Alzheimer Cognitive Composite; CF, Category verbal fluency; Trail A and Trail B, Trail Making Test Parts A and B respectively; SUVR, standardized uptake value ratio

### Image acquisition

All subjects underwent 3 T MRI using T1-weighted, resting-state fMRI, ^18^F-florbetapir PET, and ^18^F-flortaucipir PET imaging. T1-weighted imaging was performed using a sagittal 3D accelerated MPRAGE sequence with full head coverage, voxel size = 1 × 1 × 1 mm^3^, field of view = 208 × 240 × 256 mm^3^, repetition time = 2300 ms and inversion time = 900 ms. fMRI was conducted using an axial echo planar imaging sequence with voxel size = 3.4 × 3.4 × 3.4 mm^3^, field of view = 220 × 220 × 163 mm^3^, repetition time = 3000 ms, echo time = 30 ms, and flip angle = 90°. ^18^F-florbetapir PET scans were acquired in 4 × 5 min frames, 50–70 min after the injection of 10 mCi dose on average. Finally, ^18^F-flortaucipir PET images were acquired following an injection of 10.0 ± 1.0 mCi dose of [^18^F]-AV1451. They were acquired for 30 min in 6 frames (5 min per frame), 75–105 min after the injection. More information about the MRI and PET acquisition methods is provided at: https://adni.loni.usc.edu/data-samples/data-types/.

### Image preprocessing

Functional and structural MRI scans were pre-processed using a standardized pipeline implemented in fMRIPrep [29] (v20.2.4, https://fmriprep.org/en/stable/). The first two volumes of the functional scans were removed to account for steady state magnetization effects. Then, functional images were motion-corrected and adjusted for slice timing effects. Brain extraction and registration using a two-stage registration approach to 2-mm resolution MNI152 standard space were performed with Freesurfer [30] and ANTs [31]. The resulting functional images additionally underwent motion correction using the Friston-24 head motion model [32] and nuisance regression for signals from the white matter and cerebrospinal fluid. Finally, volumes underwent high-pass filtering with a 0.01 Hz cutoff.

We used the scalar standard uptake value ratios (SUVR) obtained from the PET scans already preprocessed using the standard ADNI pipeline. A detailed description of the PET preprocessing methods is available at https://adni.loni.usc.edu/methods/pet-analysis-method/pet-analysis/. In short, the 5-min PET frames were co-registered, averaged, and co-registered to the T1-weighted MRI images of each participant. Finally, normalized SUVR maps were created by using the whole cerebellum as a reference region [33].

### Calculation of whole-brain functional connectivity networks

**Fig. 1:**
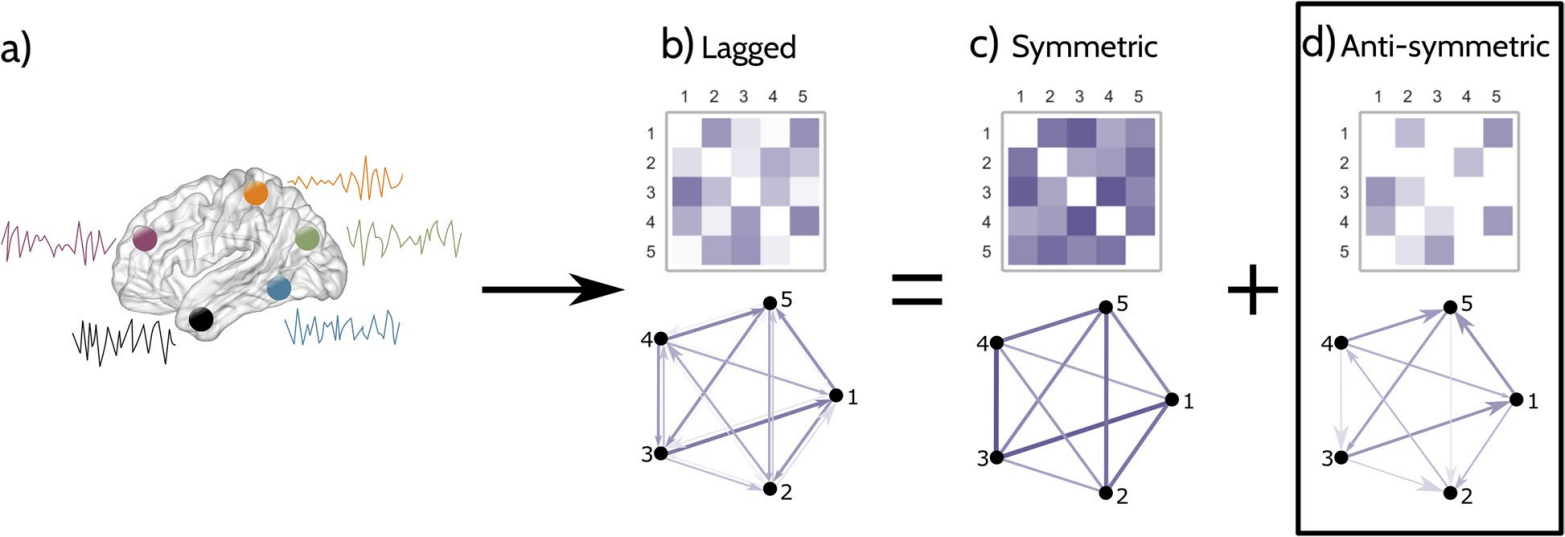
Calculation of anti-symmetric functional networks. **a** As an example, we show the time activation series of 5 brain regions represented as nodes in the brain. **b** First, we calculate delayed correlation networks by computing the delayed Pearson’s correlation coefficient between all pairs of regions at varying delays; here, the network and the delayed connectivity matrix are shown at a delay of 1. Then, we split this delayed matrix into its **c** symmetric and **d** anti-symmetric component matrices. In our subsequent analyses, we use the anti-symmetric matrices as a representation of the whole-brain-directed functional connectivity. For all matrices and networks, darker colors and thicker lines represent stronger connections

We used the temporal delay between the activity time series of two regions to define the direction and strength of the functional connection between them. The process for calculating the connectivity strength using this method is illustrated in Fig. 1, for an example of five brain regions and their corresponding activation time series (Fig. 1a). First, we calculated the delayed correlation connectivity matrix, where each entry represents the Pearson’s correlation coefficient between the time series of the corresponding brain regions after a given time delay (Fig. 1b). This delay is expressed as the number of time steps (where one-time step corresponds to one fMRI repetition time) by which one-time series is shifted relative to the other while calculating the corresponding correlation coefficient. This shift also determines the direction of the interregional connection, with earlier activated brain regions being the source and the later activated brain regions being the end of the connection.

Since it is a square matrix, this delayed correlation matrix can be univocally split into the sum of a symmetric matrix (Fig. 1c) and an anti-symmetric matrix (Fig. 1d). The anti-symmetric matrix identifies the directed connections between brain areas, capturing the directionality of the functional network. We summarize the whole-brain-directed functional connectivity of each participant using this anti-symmetric matrix, which represents a topological map of the functional network at different temporal delays. Networks calculated at short time delays represent the patterns of connectivity between brain regions that are topologically close and have strong, direct connections. On the contrary, networks calculated at higher delays capture the connectivity between regions via indirect paths of varying lengths. As the anti-symmetric correlations can evaluate functional connectivity over a wide range of temporal delays, they can be used to study the organization of brain activity patterns at different levels of topological connectivity [22].

### Network construction

Using the anti-symmetric correlation method described above, we calculated the edges of a weighted connectivity network for each participant, with the network nodes corresponding to the 200 brain regions derived from the Craddock atlas [34]. For each weighted network, we computed a set of binary networks by assigning a value of 1 for each correlation coefficient that was above a certain threshold and 0 otherwise. Since currently there is no consensus regarding which threshold should be used [25], we performed the thresholding taking into account a wide range of network densities (D_min_ = 5% to D_max_ = 50% in steps of 1%). We did not consider densities below 5% since most nodes were disconnected at such low densities. The negative correlation coefficients were also set to zero.

We calculated the area under the curve (AUC) to summarize the behavior of the network measures across the complete density range, since it is less affected by the thresholding process and does not require selecting a specific density for the correlation analysis [25]. We estimated the AUC for each network measure by numerically integrating the measure values across the complete network density range. This resulted in a single numerical value for each network measure at each temporal delay that was then used in the between-group comparisons as well as in the linear modeling.

### Network analysis

**Fig. 2:**
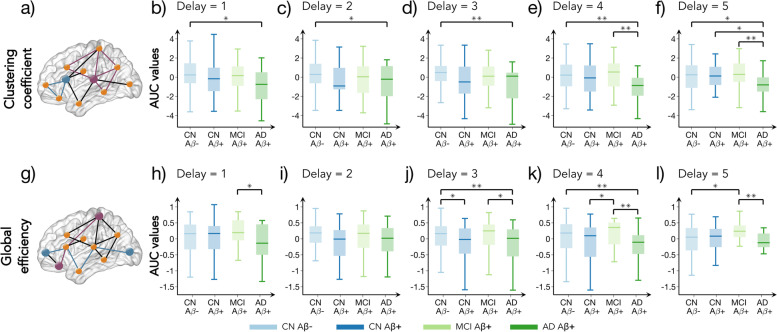
Global network measures as a function of temporal delay across the AD continuum. **a** Schematic illustration of the clustering coefficient. The purple node has a high clustering coefficient when compared to the blue node as it has a higher number of triangles around it. The black connections do not contribute to the calculation as they are not part of a closed triangle. **b**–**f** The AUC values of clustering coefficient for all groups at delays 1–5 respectively, calculated in the density range 5–50%. **g** Schematic illustration of global efficiency. The purple nodes have larger global efficiency when compared to blue nodes as they are more directly connected or through shorter paths. **h**–**l** The AUC values of global efficiency (density range: 5–50%) at temporal delays 1–5. In all plots, the boxplots denote the 25th and 75th percentiles of the data, while the whiskers extend to the largest and smallest data points. * Indicates a statistically significant result at *p* < 0.05. ** Indicates a statistically significant result at *p* < 0.001. All results were corrected for multiple comparisons using FDR

We used two network measures, namely the clustering coefficient and the global efficiency, to assess the global topology of the individual connectivity networks. The clustering coefficient (illustrated in Fig. 2a) is a measure of network segregation that is calculated as the fraction of closed triangles around a node and increases with the number of local connections. In contrast, the global efficiency (illustrated in Fig. 2g) reflects the level of network integration and it increases when the paths connecting any two nodes in the network become shorter. All graph measures were calculated using the Brain Analysis using Graph Theory software [35] (BRAPH, http://braph.org/).

### Statistical analysis

The statistical significance of the differences between different groups was assessed by performing nonparametric permutation tests with 10,000 replicates, which were considered significant for a two-tailed test of the null hypothesis at *p* < 0.05. To assess whether the functional network measures were associated with pathology and cognition, we ran linear regression models across all Aß positive individuals using the global Aß and tau Braak stages I–II, III–IV, and V–VI SUVR values, as well as cognitive test scores, as dependent variables in separate models for each variable. In these analyses, we only included subjects with values within the 1.5 interquartile range (to avoid the influence of outliers) and included cognitive status as a covariate. We built a separate linear model for each temporal delay, including age, sex, education, cognitive status, and the AUC values for the two network measures as independent variables (Supplementary Table S3). The best model was chosen as a combination of predictors that resulted in the minimum value of the Akaike information criterion (AIC). We report the best-fitting model (full model and individual predictor significance as well as the adjusted *R*^2^) for each dependent variable that included the network measures as significant predictors. The significance of the overall model and the independent coefficients was evaluated by a *F*-test, which was considered significant at *p* < 0.05. These results were adjusted for multiple comparisons by applying false discovery rate (FDR) corrections at* q* < 0.05 using the Benjamini–Hochberg procedure [36] to control for the number of clinical tests, pathology, and different temporal delays.

## Results

To identify the most relevant temporal delays for characterizing functional connectivity changes in AD, we calculated a representative network for each group by averaging the individual weighted connectivity matrices. The histograms of the connectivity weights in these group-average networks, included in Supplementary Fig. S1, show that all groups exhibit narrower strength distributions as the temporal delays increase. This agrees with a previous study [22] and suggests that using longer temporal delays results in networks containing many connections with similar functional strengths, making them unsuitable for our analysis as they fail to detect any changes in the directed functional flow. Therefore, we limited our analysis to temporal delays ranging from one to five.

### The clustering of nearby connections is increased in later stages of AD

The clustering coefficient did not show any significant differences between CN Aß + or MCI Aß + compared to CN Aß − individuals (Fig. 2b–f). However, the AD Aß + group showed a widespread decrease in the clustering coefficient at all temporal delays when compared to the CN Aß- group (Fig. 2b–f). The AD Aß + group also had lower clustering coefficient at high temporal delays when compared to the other Aß + groups. These decreases were most pronounced at temporal delays 4 and 5 in comparison to MCI Aß + (Fig. 2e, f), and at delay 5 in comparison to CN Aß + (Fig. 2f). More details about these comparisons are shown in Supplementary Table S1.

### The efficiency of direct connections shows nonlinear changes across the AD continuum

The global efficiency of CN Aß + individuals decreased at delay 3 compared to the CN Aß − group (Fig. 2j and Supplementary Table S2). Furthermore, the MCI Aß + group showed increased functional integration at delays 4 and 5 when compared to both the CN Aß + and CN Aß- groups (Fig. 2k and l, respectively). However, these increases were followed by an efficiency decrease in AD Aß + at multiple delays when compared to the MCI Aß + group (Fig. 2h, j, k, l).

### Network measures show nonlinear association with Aß and tau pathology as well as cognition

**Fig. 3:**
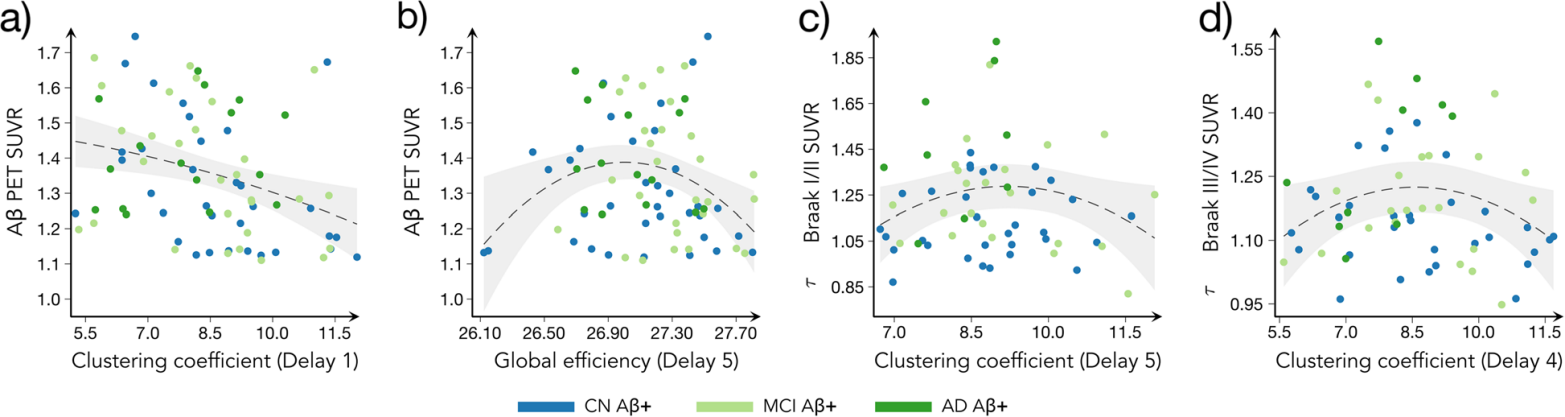
Correlation between global network measures with amyloid and tau pathology. Correlation plots showing the linear models with the significant relationships between the clustering coefficient and global efficiency with global Aβ PET SUVR values (**a**, **b**) and tau Braak SUVR (**c**, **d**) values, while controlling for age, sex, education, and cognitive status. To account for the nonlinear relationships, the squares of the network measures were also included in the model. The gray areas show the 95% confidence intervals (CI) for the predictions, whereas the dashed lines show the best model fit. The dots correspond to show CN Aβ + (blue), MCI Aβ + (light green), and AD Aβ + (dark green) subjects. All results were corrected for multiple comparisons using FDR

To identify which measures were associated with brain pathology, we fitted separate linear models across the AD continuum (all Aβ + subjects), including global Aβ-PET and tau-PET Braak I–II, III–IV, and V–VI SUVR values as dependent variables. The linear models tested for nonlinear associations of second order between pathological variables and network measures at each temporal delay separately, while controlling for age, sex, education and cognitive status (see the section “Statistical analysis”). Our findings revealed a quadratic association between brain pathology and network measures (Fig. 3), indicating that both amyloid and tau burden exhibited an initial increase with higher network measures. However, even higher levels of global efficiency and clustering coefficient were afterwards associated with a reduction in brain pathology. Specifically, lower Aβ-PET burden was associated with higher clustering coefficient at delay 1 (*p*-value = 0.003, *R*^2^ = 0.095) and higher global efficiency at delay 5 (*p*-value = 0.014, *R*^2^ = 0.083). Regarding tau-PET burden, we found that lower Braak stage I/II values and Braak III/IV values were associated with higher clustering coefficient at delay 5 (*p*-value < 0.001, *R*^2^ = 0.239) and at delay 4 (*p*-value = 0.005, *R*^2^ = 0.171), respectively.

These findings remained unchanged after adding the time interval between the functional and PET scans as an additional covariate.

**Fig. 4:**
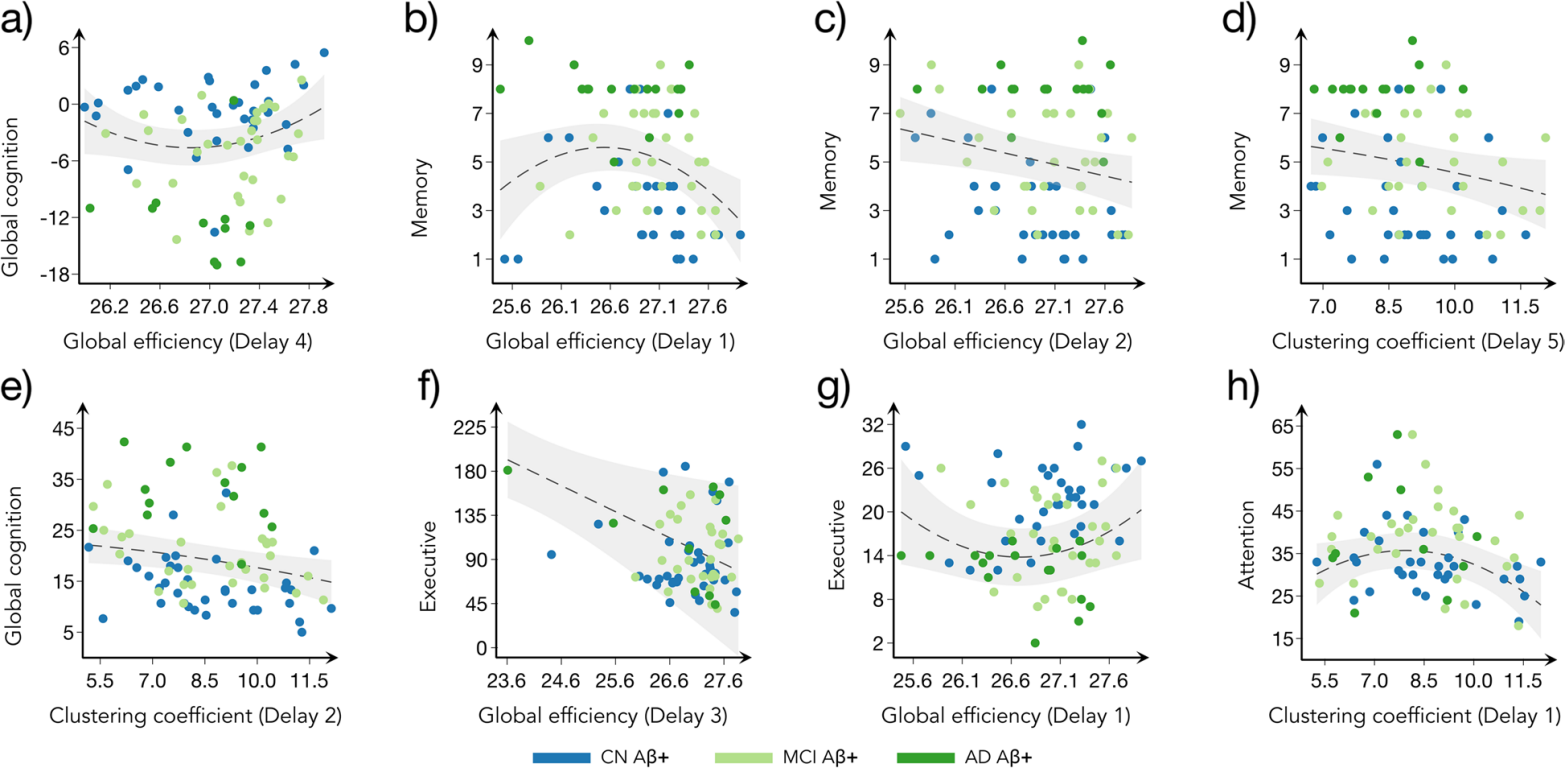
Correlation between global network measures and cognitive test scores. Plots showing the linear models between **a** global cognition (measured by mPACCtrailsB) and global efficiency at delay 4; **b**, **c** memory (measured by ADAS Q4) and global efficiency at delays 1 and 2 respectively and **d** clustering coefficient at delay 5; **e** global cognition (measured by ADAS13) and clustering coefficient at delay 2; **f** executive function (measured by Trail Making Test—Part B) and global efficiency at delay 3; **g** executive function (measured by Category Fluency (Animals)) and global efficiency at delay 1; **h** attention (measured by Trail Making Test—Part A) and clustering coefficient at delay 1. Each model includes cognition scores as the dependent variables, the network measures as predictors, while controlling for age, sex, education, and cognitive status. To account for the nonlinear relationships, the squares of the network measures were also included in the model. The gray areas show the 95% confidence intervals (CI) for the predictions and the dashed lines show the best fit. The dots correspond to show CN Aβ + (blue), MCI Aβ + (light green), and AD Aβ + (dark green) subjects. All results were corrected for multiple comparisons using FDR

Similar non-linear quadratic relationships were observed between several cognitive tests and network measures (Fig. 4). In particular, after showing an initial decrease, better mPACC performance was associated with increased global efficiency at delay 4 (*p*-value < 0.001, *R*^2^ = 0.595). Similar relationships and an eventual decrease in ADASQ4 scores were observed with increasing global efficiency at delay 1 (*p*-value < 0.001, *R*^2^ = 0.515), as well as increasing clustering coefficient at delay 5 (*p*-value < 0.001, *R*^2^ = 0.468), while an eventual decrease in the ADAS 13 scores was also associated with increasing clustering coefficient at delay 2 (*p*-value < 0.001, *R*^2^ = 0. 556). Moreover, linear associations were observed between worse performance on the ADASQ4 test and reduced global efficiency at delay 2 (*p*-value < 0.001, *R*^2^ = 0.481). Regarding cognitive tests measuring executive function and attention, performances on the CF and trail-making test A showed initial decrease, but both eventually increased with increasing global efficiency (*p*-value < 0.001, *R*^2^ = 0. 217, and *p*-value = 0.002, *R*^2^ = 0. 185, respectively). Finally, high global efficiency was linearly associated with better performance on the trail-making test B (*p*-value < 0.001, *R*^2^ = 0. 304).

### Effect of APOE ε4 gene on delayed functional network topology

To assess the impact of the APOE ε4 gene on functional network organization, we included the number of copies of the APOE ε4 allele as an additional covariate in our analyses (Table 1). After controlling for APOE ε4, the majority of the between-group comparisons maintained their significance. The comparisons between CN Aß − and AD Aß + groups (in clustering coefficient at delay 2) and between CN Aß + and MCI Aß + groups (global efficiency at delay 4) became non-significant. However, the overall trend and direction of the changes remained consistent (Fig. 2 and Supplementary Tables S1 and S2). While all correlations with brain pathology variables remained significant, the correlations between global cognition and global efficiency at delay 4 (Fig. 4a), as well as between memory and clustering coefficient at delay 5 (Fig. 4d), lost their significance, suggesting that APOE gene plays an important role in maintaining certain cognitive abilities.

## Discussion

Closely related to behavior and cognitive performance [37], functional connectivity reflects the relationships between the activation time series measured from different brain regions [18]. These associations are commonly assessed using methods that assume that brain areas become activated at the same time [17]. However, mounting evidence shows that the activation of brain regions is not always simultaneous and some regions become activated first and are followed by the later activation of others [19–22, 38]. Incorporating this information is necessary to better understand the functional connectivity patterns occurring in AD.

In this study, we adopted a novel approach, called anti-symmetric correlations, that integrates the information contained in the temporal delays between activation of different regions to characterize the direction and strength of the functional connections. Our findings show that these anti-symmetric correlations can detect changes in the organization of functional networks that occur at multiple timescales across different stages of AD. Furthermore, they follow a nonlinear trajectory throughout the AD spectrum. In particular, we observed this nonlinear behavior in the global efficiency, which started with decreases in Aß + cognitively normal individuals, followed by increases in individuals with MCI, and ending with a strong decrease in individuals with AD. However, the clustering coefficient did not follow a similar pattern and instead showed widespread decreases only in individuals in late AD stages, suggesting that functional integration is a more sensitive indicator of network changes in AD when compared to functional segregation. The between-group differences indicated that the temporal delay needed to classify a group of individuals based on their clinical diagnosis varied depending on their disease stage across the AD spectrum. For example, alterations in functional connectivity networks between cognitively normal individuals with and without Aβ pathology were detectable only at a delay of 3, whereas longer time delays were required to identify changes present in individuals at late disease stages. Finally, both functional integration and segregation measures were associated with amyloid and tau pathology, as well as cognition, demonstrating their relevance in clinical settings.

Studies have proposed that the abnormal accumulation of Aβ in the brain is one of the earliest events in AD, which promotes a cascade of downstream processes ultimately resulting in cognitive decline and dementia [1–3]. Therefore, identifying the alterations occurring in cognitively normal individuals with Aβ burden is crucial to understand the mechanisms that initiate AD [39]. Functional MRI could be useful to detect these early changes, since it is sensitive to early synaptic dysfunction due to accumulating protein aggregates even when neurodegeneration has not yet occurred [40]. A possible mechanism for the disruption of synaptic transmission is the presence of soluble amyloid ß oligomers, which appear in the intracellular space well before any plaques can be detected and affect neural transmission on the pre- and postsynaptic side as well, eventually leading to dendritic and synaptic loss [41]. These oligomers also seem to show specific effects on glutamatergic transmission by affecting mechanisms of long-term potentiation and depression [42]. The delayed functional connectivity, of approximately 7 s, might be more sensitive to such early changes in synaptic transmission as it can evaluate downstream, polysynaptic information transfer, where these effects possibly accumulate and appear in a more widespread manner.

Our delayed functional connectivity method showed that CN Aβ + individuals had an abnormal global network topology at delay 3, characterized by a lower global efficiency compared to cognitively normal individuals without Aβ burden. This decrease indicates a lower ability of the network of CN Aβ + individuals to facilitate a quick transfer of information across different brain areas, in agreement with earlier studies identifying a reduction in neuronal activity in predementia AD stages [43–45]. By interpreting the temporal delay between brain regions as a sign of their topological proximity [19, 21, 22], our results indicate that the global efficiency decreases in the CN Aβ + group are related to the disruption in the communication between brain areas either connected directly or only through a few network connections. Such direct connections are typically established by the central regions in brain networks that play a vital role in facilitating whole-network communication [46]. Therefore, our findings show that the early functional alterations in AD occur due to the preferential spatial distribution of Aß pathology across distant and central brain regions [13, 45, 47, 48]. Since such changes were not observed at larger delays, our study demonstrates that measures that capture network-wide effects are less sensitive to detect changes in network organization in AD. Instead, measures assessing direct interregional connections and their organization are the most sensitive to changes to whole-brain connectivity changes in preclinical AD and should be considered by future studies.

Regarding middle stages of the AD continuum, individuals with MCI had an increased global efficiency compared to the CN Aβ + group at high temporal delays. This indicates that there are network-wide changes in functional connectivity in the MCI group, which could occur as a consequence of the widespread regional Aβ pathology in individuals with MCI [3]. This increased network integration could be seen as a compensatory mechanism in response to the continuing accumulation of amyloid-β [5, 12, 13], as similar results have been found in previous studies in individuals with MCI performing different cognitive tasks [9–11, 49]. However, this enhanced network integration could also have negative effects and impair the ability of the brain to process information [22, 50], which could predict a faster cognitive decline in individuals with MCI [15, 51].

After detecting hyper-global efficiency in the MCI Aβ + group, anti-symmetric correlations revealed a subsequent decrease in functional integration among individuals with AD dementia. This suggests that the observed nonlinear trajectories are probably due to the compounded effects of amyloid and tau on the functional connectivity. This interpretation is supported by the finding that the accumulation of tau and amyloid in the brain is enhanced by increased neural activity [52, 53]. Specifically, the hyperactivity that results from the initial amyloid deposition could lead to higher levels of tau, which could cause an ongoing cycle of tau and amyloid buildup [5]. Consequently, having the high deposition levels of tau and amyloid observed in AD [1] could result in disruptions in the network organization of functional connections and a decline in the functional connectivity in AD [9–11, 54].

In contrast, between-group differences were harder to detect in the clustering coefficient. We found that the clustering coefficient was lower in individuals with AD dementia in comparison to both amyloid-positive groups at high temporal delays and compared to Aβ − cognitively normal individuals at all temporal delays. The clustering coefficient measures the density of local connections and can be used as an indicator of a network’s ability to perform specialized processing tasks [25]. Combined with the reduced global efficiency observed in AD dementia patients across various temporal delays, our findings indicate a widespread disruption in the temporal organization of the functional connectivity networks in AD patients. This organization is usually called small-world and corresponds to a balance between locally clustered connections and high functional integration [25, 35]. The disruption in this organization results in a worse ability of the network to function normally and can potentially explain the severe cognitive deficits observed in AD [55–57].

Furthermore, the differences between the amyloid-positive groups along the AD continuum were observed only at higher temporal delays. As such high delays can occur between brain regions that are topologically distant from each other [19, 21, 22], these differences suggest that an increase in amyloid burden is linked to more severe network-wide disruptions (distant brain regions) that hinder the communication between brain regions through indirect connections. Therefore, our findings support the characterization of AD as a disconnection syndrome [58], demonstrating that this disconnection arises from disruptions in long-distance functional connectivity [55, 59].

We assessed the clinical relevance of the network measures by testing their associations with the amount of brain pathology and scores on cognitive tests commonly used in clinical practice to assess AD patients. The network measures were associated with both global amyloid and tau Braak stages, following a nonlinear, inverted-U pattern, in agreement with earlier studies showing that network structure correlates with amyloid and tau pathology [60, 61]. Furthermore, high network values were linked to better performance on tests measuring memory, attention, executive function, and global cognition. This is in line with previous reports showing an association between higher cognitive performance and networks with strong functional specialization and integration properties [62, 63]. As these tests are frequently used in clinical trials to evaluate the effectiveness of antidementia treatments [27, 28], our findings suggest that changes in directed network activation patterns could be a viable biomarker for tracking clinical progression in AD.

Our results should be interpreted in light of the limitation that they were obtained from cross-sectional functional MRI data. This cross-sectional design did not allow us to determine whether measures of functional segregation and integration can predict the progression of AD or the rate of amyloid or tau accumulation over time. Therefore, further studies are needed to assess these questions and examine the causal relations between these variables.

## Conclusions

In this study, we demonstrate that a functional connectivity method that uses temporal delays in the activation between brain regions can identify novel network changes in individuals at various stages of AD. Our results indicate that functional integration measures, which exhibit a nonlinear inverse-U trajectory across the AD spectrum, are more effective in detecting these differences than functional segregation measures and could explain the results obtained by previous studies showing nonlinear functional changes in AD. Furthermore, both functional integration and segregation measures had nonlinear relationships with amyloid and tau burden in the brain, as well as measures of memory and general cognitive performance. These findings suggest that this method may provide a deeper understanding of functional connectivity changes in patients at different stages of AD, as well as help improve AD diagnosis with non-invasive imaging measures.

## Supplementary Information

**Additional file 1: Supplementary Figure S1.** Connectivity strength distribution at different temporal lags. **Supplementary Table S1.** Significant between-group differences in clustering coefficient evaluated at individual network densities. **Supplementary Table S2.** Significant between-group differences in global efficiency evaluated at individual network densities. **Supplementary Table S3.** Variance Inflation Factors (VIF) between network measures as different temporal delays and age, sex, education and cognitive status.

## Data Availability

The authors did not participate in data collection. The data used in the current study were obtained from ADNI, an open-access multicenter cohort, which requires application for access (https://adni.loni.usc.edu/data-samples/access-data/). The BRAPH software can be freely downloaded from: http://braph.org/.

## Abbreviations

AD: Alzheimer’s disease
Aß: Amyloid-beta
MCI: Mild cognitive impairment
fMRI: Functional magnetic resonance imaging
PET: Positron emission tomography
ADNI: Alzheimer’s Disease Neuroimaging Initiative
MMSE: Mini-Mental State Examination
CDR-SB: Clinical Dementia Rating-Sum of Boxes
ADAS-Cog 13: Alzheimer’s Disease Assessment Scale–Cognitive Subscale
ADAS Q4: Delayed Word Recall test
mPACCtrailsB: Modified Preclinical Alzheimer Cognitive Composite with Trails test
CF: Category fluency animal naming (CF)
Trail A: Trail making test part A
Trail B: Trail making test part B
CN Aβ −: Aβ-negative cognitively normal
CN Aβ +: Aβ-positive cognitively normal
MCI Aβ +: Aβ-positive patients with mild cognitive impairment
AD Aβ +: Aβ-positive patients with AD dementia
SUVR: Standard uptake value ratios
AUC: Area under the curve
