# Supplementary material for: Nonlinear changes in delayed functional network topology in Alzheimer’s disease: relationship with amyloid and tau pathology
Source: Alzheimers Res Ther. 2023 Jun 16;15:112. doi: 10.1186/s13195-023-01252-3 (PMC10273754; doi:10.1186/s13195-023-01252-3)
Supplement: Supplementary file 1 — Additional file 1: Supplementary Figure S1. Connectivity strength distribution at different temporal lags. Supplementary Table S1. Significant between-group differences in clustering coefficient evaluated at individual network densities. Supplementary Table S2. Significant between-group differences in global efficiency evaluated at individual network densities. Supplementary Table S3. Variance Inflation Factors (VIF) between network measures as different temporal delays and age, sex, education and cognitive status. [file 13195_2023_1252_MOESM1_ESM.docx]

**Nonlinear changes in delayed functional network topology in Alzheimer’s disease: relationship with amyloid and tau pathology**

Mite Mijalkov^1*^, Dániel Veréb^1^, Anna Canal-Garcia^1^, Thomas Hinault^2^, Giovanni Volpe^3#^, Joana B Pereira^1*#^, and for the Alzheimer’s Disease Neuroimaging Initiative†

^1^ Department of Clinical Neuroscience, Karolinska Institutet, Stockholm, Sweden

^2^ Normandie Univ, Unicaen, PSL, Université Paris, EPHE, Inserm, U1077, CHU de Caen, Centre Cyceron, 14000 Caen, France

^3^ Department of Physics, Goteborg University, Goteborg, Sweden

* Corresponding authors:

Mite Mijalkov ([mite.mijalkov@ki.se](mailto:mite.mijalkov@ki.se)) // Joana B. Pereira ([joana.pereira@ki.se](mailto:joana.pereira@ki.se))

Department of Clinical Neuroscience, Karolinska Institutet, Stockholm, Sweden.

# Contributed equally as senior authors.

† Data used in preparation of this article were obtained from the Alzheimer’s Disease Neuroimaging Initiative (ADNI) database (adni.loni.usc.edu). As such, the investigators within the ADNI contributed to the design and implementation of ADNI and/or provided data but did not participate in analysis or writing of this report. A complete listing of ADNI investigators can be found at: https://adni.loni.usc.edu/wp-content/uploads/how_to_apply/ADNI_Acknowledgement_List.pdf

**Supplementary Figure 1: Connectivity strength distribution at different temporal lags.**


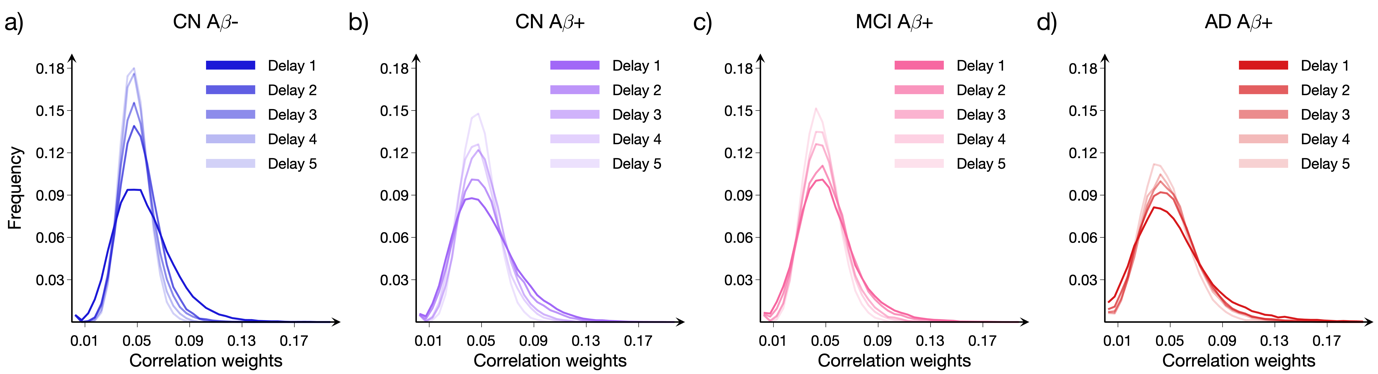


The histograms show the distribution in connectivity strengths of the average adjacency matrices for a) CN Aβ-, b) CN Aβ+, c) MCI Aβ+, and d) AD Aβ+ as a function of different temporal delays. The individual connectivity matrices were calculated using the anti-symmetric method. Only the delays used in the analysis (1 to 5) are shown in this figure; lighter colors represent higher delays. CN, cognitively normal; MCI, mild cognitive impairment; AD, Alzheimer’s disease; Aβ, amyloid-β status (positive, +; or negative, -).

**Supplementary Table S1. Significant between-group differences in clustering coefficient evaluated at individual network densities.**

| CN Aβ- vs. AD Aβ+ | | | | |  |
| --- | --- | --- | --- | --- | --- |
|  |  |  |  |  |  |
| Delay 1 | | | | |  |
|  |  |  |  |  |  |
| Density (%) | Difference | p-value | CI-low | CI-high |  |
| 10 | -0,014 | 0,047 | -0,011 | 0,012 |  |
| 11 | -0,016 | 0,041 | -0,013 | 0,012 |  |
| 12 | -0,016 | 0,036 | -0,013 | 0,013 |  |
| 13 | -0,018 | 0,03 | -0,013 | 0,013 |  |
| 14 | -0,018 | 0,037 | -0,014 | 0,014 |  |
| 15 | -0,019 | 0,032 | -0,015 | 0,014 |  |
| 16 | -0,02 | 0,023 | -0,014 | 0,015 |  |
| 17 | -0,02 | 0,025 | -0,015 | 0,015 |  |
| 18 | -0,022 | 0,022 | -0,016 | 0,016 |  |
| 19 | -0,022 | 0,023 | -0,016 | 0,016 |  |
| 20 | -0,022 | 0,022 | -0,016 | 0,016 |  |
| 21 | -0,023 | 0,018 | -0,017 | 0,016 |  |
| 22 | -0,024 | 0,021 | -0,017 | 0,016 |  |
| 23 | -0,024 | 0,019 | -0,017 | 0,017 |  |
| 24 | -0,025 | 0,019 | -0,018 | 0,017 |  |
| 25 | -0,025 | 0,018 | -0,018 | 0,018 |  |
| 26 | -0,026 | 0,017 | -0,018 | 0,018 |  |
| 27 | -0,026 | 0,02 | -0,019 | 0,019 |  |
| 28 | -0,026 | 0,022 | -0,019 | 0,018 |  |
| 29 | -0,027 | 0,023 | -0,02 | 0,019 |  |
| 30 | -0,027 | 0,019 | -0,019 | 0,019 |  |
| 31 | -0,027 | 0,021 | -0,02 | 0,02 |  |
| 32 | -0,028 | 0,019 | -0,02 | 0,019 |  |
| 33 | -0,028 | 0,02 | -0,021 | 0,02 |  |
| 34 | -0,029 | 0,016 | -0,021 | 0,019 |  |
| 35 | -0,029 | 0,02 | -0,021 | 0,021 |  |
| 36 | -0,029 | 0,017 | -0,021 | 0,02 |  |
| 37 | -0,03 | 0,015 | -0,021 | 0,021 |  |
| 38 | -0,03 | 0,016 | -0,021 | 0,02 |  |
| 39 | -0,03 | 0,015 | -0,021 | 0,02 |  |
| 40 | -0,03 | 0,016 | -0,021 | 0,02 |  |
| 41 | -0,03 | 0,013 | -0,021 | 0,021 |  |
| 42 | -0,03 | 0,015 | -0,021 | 0,021 |  |
| 43 | -0,03 | 0,015 | -0,021 | 0,02 |  |
| 44 | -0,03 | 0,018 | -0,022 | 0,02 |  |
| 45 | -0,03 | 0,017 | -0,021 | 0,021 |  |
| 46 | -0,03 | 0,018 | -0,021 | 0,021 |  |
| 47 | -0,03 | 0,016 | -0,021 | 0,02 |  |
| 48 | -0,029 | 0,015 | -0,02 | 0,02 |  |
| 49 | -0,03 | 0,011 | -0,02 | 0,02 |  |
| 50 | -0,029 | 0,016 | -0,02 | 0,02 |  |
| Delay 2 | | | | |  |
|  |  |  |  |  |  |
| Density (%) | Difference | p-value | CI-low | CI-high |  |
| 9 | -0,012 | 0,048 | -0,01 | 0,01 |  |
| 16 | -0,017 | 0,043 | -0,014 | 0,014 |  |
| 17 | -0,018 | 0,036 | -0,014 | 0,014 |  |
| 18 | -0,018 | 0,033 | -0,014 | 0,014 |  |
| 19 | -0,019 | 0,031 | -0,015 | 0,015 |  |
| 20 | -0,02 | 0,03 | -0,015 | 0,015 |  |
| 21 | -0,02 | 0,03 | -0,015 | 0,015 |  |
| 22 | -0,021 | 0,031 | -0,016 | 0,015 |  |
| 23 | -0,021 | 0,035 | -0,016 | 0,016 |  |
| 24 | -0,022 | 0,029 | -0,017 | 0,016 |  |
| 25 | -0,021 | 0,037 | -0,017 | 0,016 |  |
| 26 | -0,021 | 0,039 | -0,017 | 0,017 |  |
| 27 | -0,022 | 0,035 | -0,018 | 0,017 |  |
| 28 | -0,022 | 0,04 | -0,017 | 0,017 |  |
| 29 | -0,022 | 0,044 | -0,018 | 0,017 |  |
| 30 | -0,022 | 0,042 | -0,019 | 0,018 |  |
| 31 | -0,022 | 0,048 | -0,019 | 0,018 |  |
| 32 | -0,022 | 0,042 | -0,019 | 0,018 |  |
| 33 | -0,022 | 0,047 | -0,019 | 0,018 |  |
| 34 | -0,023 | 0,045 | -0,019 | 0,019 |  |
| 35 | -0,023 | 0,048 | -0,02 | 0,018 |  |
| 36 | -0,023 | 0,041 | -0,019 | 0,019 |  |
| 37 | -0,023 | 0,049 | -0,02 | 0,019 |  |
| 38 | -0,024 | 0,047 | -0,02 | 0,019 |  |
| 39 | -0,024 | 0,038 | -0,019 | 0,019 |  |
| 40 | -0,023 | 0,043 | -0,019 | 0,018 |  |
| 41 | -0,024 | 0,042 | -0,02 | 0,018 |  |
| 42 | -0,024 | 0,044 | -0,02 | 0,019 |  |
| 43 | -0,023 | 0,039 | -0,019 | 0,018 |  |
| 44 | -0,024 | 0,045 | -0,02 | 0,019 |  |
| 45 | -0,023 | 0,043 | -0,02 | 0,018 |  |
| 46 | -0,023 | 0,041 | -0,019 | 0,019 |  |
| 47 | -0,023 | 0,041 | -0,019 | 0,018 |  |
| 48 | -0,022 | 0,044 | -0,019 | 0,018 |  |
| 49 | -0,023 | 0,039 | -0,019 | 0,017 |  |
| 50 | -0,023 | 0,04 | -0,019 | 0,017 |  |
| Delay 3 | | | | |  |
|  |  |  |  |  |  |
| Density (%) | Difference | p-value | CI-low | CI-high |  |
| 5 | -0,009 | 0,022 | -0,007 | 0,007 |  |
| 6 | -0,01 | 0,029 | -0,007 | 0,008 |  |
| 7 | -0,011 | 0,027 | -0,008 | 0,008 |  |
| 8 | -0,012 | 0,022 | -0,009 | 0,009 |  |
| 9 | -0,013 | 0,019 | -0,009 | 0,01 |  |
| 10 | -0,014 | 0,024 | -0,01 | 0,01 |  |
| 11 | -0,015 | 0,02 | -0,011 | 0,011 |  |
| 12 | -0,015 | 0,029 | -0,011 | 0,011 |  |
| 13 | -0,016 | 0,022 | -0,011 | 0,011 |  |
| 14 | -0,017 | 0,02 | -0,012 | 0,012 |  |
| 15 | -0,017 | 0,024 | -0,012 | 0,012 |  |
| 16 | -0,017 | 0,022 | -0,013 | 0,012 |  |
| 17 | -0,019 | 0,018 | -0,013 | 0,013 |  |
| 18 | -0,019 | 0,016 | -0,013 | 0,013 |  |
| 19 | -0,019 | 0,017 | -0,014 | 0,014 |  |
| 20 | -0,021 | 0,015 | -0,014 | 0,014 |  |
| 21 | -0,021 | 0,018 | -0,015 | 0,014 |  |
| 22 | -0,022 | 0,013 | -0,015 | 0,014 |  |
| 23 | -0,023 | 0,011 | -0,015 | 0,015 |  |
| 24 | -0,024 | 0,009 | -0,015 | 0,015 |  |
| 25 | -0,025 | 0,01 | -0,016 | 0,015 |  |
| 26 | -0,025 | 0,008 | -0,016 | 0,016 |  |
| 27 | -0,026 | 0,008 | -0,016 | 0,016 |  |
| 28 | -0,026 | 0,008 | -0,017 | 0,016 |  |
| 29 | -0,027 | 0,007 | -0,017 | 0,016 |  |
| 30 | -0,027 | 0,007 | -0,017 | 0,016 |  |
| 31 | -0,028 | 0,005 | -0,018 | 0,017 |  |
| 32 | -0,028 | 0,006 | -0,018 | 0,017 |  |
| 33 | -0,028 | 0,008 | -0,018 | 0,017 |  |
| 34 | -0,029 | 0,007 | -0,018 | 0,017 |  |
| 35 | -0,029 | 0,006 | -0,018 | 0,017 |  |
| 36 | -0,029 | 0,008 | -0,018 | 0,017 |  |
| 37 | -0,03 | 0,005 | -0,018 | 0,018 |  |
| 38 | -0,03 | 0,005 | -0,019 | 0,018 |  |
| 39 | -0,03 | 0,004 | -0,018 | 0,018 |  |
| 40 | -0,031 | 0,004 | -0,018 | 0,018 |  |
| 41 | -0,031 | 0,004 | -0,019 | 0,017 |  |
| 42 | -0,03 | 0,005 | -0,018 | 0,017 |  |
| 43 | -0,03 | 0,005 | -0,018 | 0,017 |  |
| 44 | -0,03 | 0,006 | -0,019 | 0,017 |  |
| 45 | -0,03 | 0,004 | -0,019 | 0,017 |  |
| 46 | -0,03 | 0,005 | -0,018 | 0,017 |  |
| 47 | -0,03 | 0,005 | -0,018 | 0,017 |  |
| 48 | -0,029 | 0,004 | -0,018 | 0,016 |  |
| 49 | -0,028 | 0,005 | -0,017 | 0,016 |  |
| 50 | -0,029 | 0,005 | -0,017 | 0,016 |  |
| Delay 4 | | | | |  |
|  |  |  |  |  |  |
| Density (%) | Difference | p-value | CI-low | CI-high |  |
| 5 | -0,011 | 0,006 | -0,007 | 0,007 |  |
| 6 | -0,013 | 0,003 | -0,007 | 0,008 |  |
| 7 | -0,013 | 0,007 | -0,008 | 0,009 |  |
| 8 | -0,015 | 0,006 | -0,009 | 0,01 |  |
| 9 | -0,016 | 0,005 | -0,009 | 0,01 |  |
| 10 | -0,017 | 0,003 | -0,01 | 0,01 |  |
| 11 | -0,018 | 0,006 | -0,01 | 0,011 |  |
| 12 | -0,02 | 0,003 | -0,011 | 0,011 |  |
| 13 | -0,021 | 0,003 | -0,012 | 0,012 |  |
| 14 | -0,022 | 0,002 | -0,012 | 0,012 |  |
| 15 | -0,022 | 0,003 | -0,012 | 0,013 |  |
| 16 | -0,022 | 0,004 | -0,013 | 0,013 |  |
| 17 | -0,024 | 0,003 | -0,013 | 0,013 |  |
| 18 | -0,024 | 0,002 | -0,014 | 0,014 |  |
| 19 | -0,026 | 0,002 | -0,014 | 0,014 |  |
| 20 | -0,027 | 0,002 | -0,014 | 0,014 |  |
| 21 | -0,027 | 0,002 | -0,015 | 0,014 |  |
| 22 | -0,028 | 0,002 | -0,015 | 0,015 |  |
| 23 | -0,029 | 0,002 | -0,016 | 0,016 |  |
| 24 | -0,03 | 0,002 | -0,016 | 0,016 |  |
| 25 | -0,03 | 0,002 | -0,016 | 0,016 |  |
| 26 | -0,031 | 0,001 | -0,016 | 0,016 |  |
| 27 | -0,032 | 0,002 | -0,017 | 0,016 |  |
| 28 | -0,032 | 0,001 | -0,017 | 0,016 |  |
| 29 | -0,032 | 0,001 | -0,017 | 0,017 |  |
| 30 | -0,033 | 0,002 | -0,018 | 0,018 |  |
| 31 | -0,034 | 0,002 | -0,018 | 0,018 |  |
| 32 | -0,034 | 0,001 | -0,018 | 0,018 |  |
| 33 | -0,035 | 0,001 | -0,018 | 0,018 |  |
| 34 | -0,035 | 0,002 | -0,019 | 0,018 |  |
| 35 | -0,035 | 0,001 | -0,019 | 0,018 |  |
| 36 | -0,036 | 0,001 | -0,019 | 0,019 |  |
| 37 | -0,036 | 0,001 | -0,019 | 0,019 |  |
| 38 | -0,036 | 0,002 | -0,019 | 0,019 |  |
| 39 | -0,036 | 0,001 | -0,019 | 0,019 |  |
| 40 | -0,036 | 0,001 | -0,02 | 0,019 |  |
| 41 | -0,036 | 0,002 | -0,019 | 0,019 |  |
| 42 | -0,036 | 0,002 | -0,019 | 0,019 |  |
| 43 | -0,035 | 0,002 | -0,019 | 0,019 |  |
| 44 | -0,036 | 0,003 | -0,019 | 0,019 |  |
| 45 | -0,035 | 0,003 | -0,019 | 0,018 |  |
| 46 | -0,035 | 0,002 | -0,02 | 0,018 |  |
| 47 | -0,035 | 0,002 | -0,019 | 0,018 |  |
| 48 | -0,034 | 0,002 | -0,019 | 0,018 |  |
| 49 | -0,034 | 0,002 | -0,018 | 0,018 |  |
| 50 | -0,033 | 0,003 | -0,018 | 0,018 |  |
| Delay 5 | | | | |  |
|  |  |  |  |  |  |
| Density (%) | Difference | p-value | CI-low | CI-high |  |
| 5 | -0,008 | 0,03 | -0,006 | 0,006 |  |
| 20 | -0,017 | 0,047 | -0,014 | 0,014 |  |
| 21 | -0,018 | 0,041 | -0,014 | 0,014 |  |
| 22 | -0,018 | 0,041 | -0,015 | 0,015 |  |
| 23 | -0,019 | 0,038 | -0,015 | 0,015 |  |
| 24 | -0,019 | 0,039 | -0,015 | 0,015 |  |
| 25 | -0,02 | 0,037 | -0,016 | 0,015 |  |
| 26 | -0,02 | 0,035 | -0,016 | 0,016 |  |
| 27 | -0,021 | 0,033 | -0,016 | 0,016 |  |
| 28 | -0,022 | 0,032 | -0,017 | 0,017 |  |
| 29 | -0,022 | 0,035 | -0,017 | 0,017 |  |
| 30 | -0,022 | 0,033 | -0,017 | 0,017 |  |
| 31 | -0,023 | 0,031 | -0,017 | 0,017 |  |
| 32 | -0,023 | 0,034 | -0,017 | 0,017 |  |
| 33 | -0,023 | 0,026 | -0,018 | 0,017 |  |
| 34 | -0,023 | 0,028 | -0,018 | 0,018 |  |
| 35 | -0,023 | 0,03 | -0,018 | 0,018 |  |
| 36 | -0,023 | 0,034 | -0,018 | 0,017 |  |
| 37 | -0,023 | 0,028 | -0,018 | 0,018 |  |
| 38 | -0,024 | 0,027 | -0,018 | 0,018 |  |
| 39 | -0,023 | 0,036 | -0,018 | 0,018 |  |
| 40 | -0,023 | 0,035 | -0,018 | 0,018 |  |
| 41 | -0,023 | 0,041 | -0,018 | 0,018 |  |
| 42 | -0,023 | 0,032 | -0,018 | 0,017 |  |
| 43 | -0,023 | 0,031 | -0,018 | 0,017 |  |
| 44 | -0,023 | 0,033 | -0,018 | 0,017 |  |
| 45 | -0,023 | 0,031 | -0,017 | 0,017 |  |
| 46 | -0,023 | 0,035 | -0,018 | 0,017 |  |
| 47 | -0,022 | 0,033 | -0,017 | 0,017 |  |
| 48 | -0,021 | 0,041 | -0,017 | 0,017 |  |
| 49 | -0,021 | 0,036 | -0,017 | 0,017 |  |
| 50 | -0,021 | 0,039 | -0,017 | 0,016 |  |
| CN Aβ- vs. CN Aβ+ | | | | |  |
|  |  |  |  |  |  |
| Delay 3 | | | | |  |
|  |  |  |  |  |  |
| Density (%) | Difference | p-value | CI-low | CI-high |  |
| 25 | -0,015 | 0,049 | -0,012 | 0,012 |  |
| 26 | -0,015 | 0,046 | -0,013 | 0,013 |  |
| 27 | -0,016 | 0,042 | -0,013 | 0,013 |  |
| 28 | -0,016 | 0,046 | -0,013 | 0,013 |  |
| 29 | -0,016 | 0,045 | -0,013 | 0,013 |  |
| 30 | -0,016 | 0,047 | -0,014 | 0,013 |  |
| 31 | -0,017 | 0,037 | -0,014 | 0,014 |  |
| 32 | -0,017 | 0,034 | -0,014 | 0,013 |  |
| 33 | -0,018 | 0,03 | -0,014 | 0,014 |  |
| 34 | -0,018 | 0,029 | -0,014 | 0,014 |  |
| 35 | -0,019 | 0,032 | -0,014 | 0,014 |  |
| 36 | -0,019 | 0,024 | -0,014 | 0,014 |  |
| 37 | -0,019 | 0,024 | -0,014 | 0,014 |  |
| 38 | -0,019 | 0,025 | -0,014 | 0,014 |  |
| 39 | -0,02 | 0,023 | -0,014 | 0,014 |  |
| 40 | -0,02 | 0,022 | -0,014 | 0,014 |  |
| 41 | -0,02 | 0,018 | -0,014 | 0,014 |  |
| 42 | -0,02 | 0,019 | -0,014 | 0,014 |  |
| 43 | -0,02 | 0,019 | -0,014 | 0,014 |  |
| 44 | -0,02 | 0,018 | -0,014 | 0,014 |  |
| 45 | -0,019 | 0,02 | -0,013 | 0,013 |  |
| 46 | -0,019 | 0,018 | -0,014 | 0,013 |  |
| 47 | -0,019 | 0,017 | -0,014 | 0,013 |  |
| 48 | -0,019 | 0,017 | -0,013 | 0,013 |  |
| 49 | -0,018 | 0,019 | -0,013 | 0,013 |  |
| 50 | -0,018 | 0,021 | -0,013 | 0,013 |  |
| CN Aβ+ vs. AD Aβ+ | | | | |  |
|  |  |  |  |  |  |
| Delay 4 | | | | |  |
|  |  |  |  |  |  |
| Density (%) | Difference | p-value | CI-low | CI-high |  |
| 5 | -0,012 | 0,006 | -0,007 | 0,008 |  |
| 6 | -0,013 | 0,019 | -0,009 | 0,009 |  |
| 7 | -0,013 | 0,027 | -0,01 | 0,01 |  |
| 8 | -0,014 | 0,027 | -0,011 | 0,011 |  |
| 9 | -0,015 | 0,033 | -0,011 | 0,012 |  |
| 10 | -0,015 | 0,036 | -0,012 | 0,012 |  |
| 12 | -0,016 | 0,043 | -0,013 | 0,014 |  |
| 13 | -0,017 | 0,041 | -0,014 | 0,014 |  |
| Delay 5 | | | | |  |
|  |  |  |  |  |  |
| Density (%) | Difference | p-value | CI-low | CI-high |  |
| 5 | -0,011 | 0,016 | -0,007 | 0,008 |  |
| 6 | -0,011 | 0,024 | -0,008 | 0,009 |  |
| 7 | -0,01 | 0,047 | -0,009 | 0,009 |  |
| 8 | -0,011 | 0,032 | -0,009 | 0,009 |  |
| 9 | -0,012 | 0,035 | -0,009 | 0,009 |  |
| 10 | -0,014 | 0,023 | -0,01 | 0,01 |  |
| 11 | -0,015 | 0,021 | -0,01 | 0,011 |  |
| 12 | -0,015 | 0,024 | -0,011 | 0,011 |  |
| 13 | -0,015 | 0,027 | -0,012 | 0,011 |  |
| 14 | -0,015 | 0,033 | -0,012 | 0,012 |  |
| 15 | -0,017 | 0,025 | -0,012 | 0,012 |  |
| 16 | -0,017 | 0,021 | -0,013 | 0,013 |  |
| 17 | -0,017 | 0,029 | -0,013 | 0,013 |  |
| 18 | -0,018 | 0,026 | -0,013 | 0,013 |  |
| 19 | -0,018 | 0,026 | -0,014 | 0,014 |  |
| 20 | -0,019 | 0,023 | -0,014 | 0,014 |  |
| 21 | -0,02 | 0,023 | -0,015 | 0,014 |  |
| 22 | -0,02 | 0,024 | -0,015 | 0,015 |  |
| 23 | -0,021 | 0,027 | -0,015 | 0,015 |  |
| 24 | -0,02 | 0,031 | -0,016 | 0,016 |  |
| 25 | -0,021 | 0,026 | -0,016 | 0,016 |  |
| 26 | -0,021 | 0,03 | -0,016 | 0,016 |  |
| 27 | -0,021 | 0,037 | -0,017 | 0,017 |  |
| 28 | -0,022 | 0,031 | -0,017 | 0,017 |  |
| 29 | -0,022 | 0,038 | -0,017 | 0,017 |  |
| 30 | -0,022 | 0,04 | -0,018 | 0,017 |  |
| 31 | -0,023 | 0,037 | -0,019 | 0,018 |  |
| 32 | -0,022 | 0,043 | -0,018 | 0,018 |  |
| 33 | -0,022 | 0,045 | -0,019 | 0,018 |  |
| 34 | -0,023 | 0,048 | -0,019 | 0,019 |  |
| MCI Aβ+ vs. AD Aβ+ | | | | |  |
|  |  |  |  |  |  |
| Delay 4 | | | | |  |
|  |  |  |  |  |  |
| Density (%) | Difference | p-value | CI-low | CI-high |  |
| 5 | -0,013 | 0,002 | -0,007 | 0,007 |  |
| 6 | -0,014 | 0,003 | -0,008 | 0,008 |  |
| 7 | -0,015 | 0,003 | -0,009 | 0,009 |  |
| 8 | -0,017 | 0,003 | -0,01 | 0,01 |  |
| 9 | -0,019 | 0,002 | -0,01 | 0,011 |  |
| 10 | -0,02 | 0,002 | -0,011 | 0,011 |  |
| 11 | -0,02 | 0,003 | -0,012 | 0,011 |  |
| 12 | -0,022 | 0,003 | -0,012 | 0,012 |  |
| 13 | -0,023 | 0,004 | -0,013 | 0,013 |  |
| 14 | -0,023 | 0,003 | -0,013 | 0,013 |  |
| 15 | -0,024 | 0,003 | -0,014 | 0,013 |  |
| 16 | -0,024 | 0,005 | -0,014 | 0,014 |  |
| 17 | -0,025 | 0,003 | -0,014 | 0,014 |  |
| 18 | -0,025 | 0,007 | -0,015 | 0,015 |  |
| 19 | -0,026 | 0,004 | -0,015 | 0,015 |  |
| 20 | -0,027 | 0,005 | -0,016 | 0,016 |  |
| 21 | -0,027 | 0,007 | -0,016 | 0,016 |  |
| 22 | -0,028 | 0,006 | -0,017 | 0,017 |  |
| 23 | -0,029 | 0,006 | -0,017 | 0,017 |  |
| 24 | -0,03 | 0,005 | -0,018 | 0,018 |  |
| 25 | -0,031 | 0,005 | -0,019 | 0,018 |  |
| 26 | -0,032 | 0,005 | -0,019 | 0,018 |  |
| 27 | -0,033 | 0,004 | -0,019 | 0,019 |  |
| 28 | -0,033 | 0,006 | -0,02 | 0,019 |  |
| 29 | -0,033 | 0,004 | -0,02 | 0,02 |  |
| 30 | -0,033 | 0,004 | -0,02 | 0,02 |  |
| 31 | -0,034 | 0,005 | -0,02 | 0,021 |  |
| 32 | -0,035 | 0,004 | -0,021 | 0,021 |  |
| 33 | -0,035 | 0,004 | -0,021 | 0,021 |  |
| 34 | -0,035 | 0,005 | -0,021 | 0,021 |  |
| 35 | -0,035 | 0,005 | -0,022 | 0,021 |  |
| 36 | -0,036 | 0,005 | -0,022 | 0,021 |  |
| 37 | -0,036 | 0,005 | -0,022 | 0,022 |  |
| 38 | -0,036 | 0,005 | -0,022 | 0,022 |  |
| 39 | -0,036 | 0,005 | -0,022 | 0,023 |  |
| 40 | -0,036 | 0,006 | -0,022 | 0,022 |  |
| 41 | -0,036 | 0,006 | -0,022 | 0,022 |  |
| 42 | -0,036 | 0,006 | -0,022 | 0,022 |  |
| 43 | -0,036 | 0,004 | -0,023 | 0,021 |  |
| 44 | -0,036 | 0,005 | -0,022 | 0,022 |  |
| 45 | -0,035 | 0,008 | -0,022 | 0,022 |  |
| 46 | -0,035 | 0,006 | -0,022 | 0,022 |  |
| 47 | -0,035 | 0,005 | -0,022 | 0,022 |  |
| 48 | -0,035 | 0,006 | -0,022 | 0,021 |  |
| 49 | -0,034 | 0,007 | -0,022 | 0,021 |  |
| 50 | -0,034 | 0,007 | -0,021 | 0,021 |  |
| Delay 5 | | | | |  |
|  |  |  |  |  |  |
| Density (%) | Difference | p-value | CI-low | CI-high |  |
| 5 | -0,013 | 0,003 | -0,008 | 0,008 |  |
| 6 | -0,014 | 0,01 | -0,009 | 0,009 |  |
| 7 | -0,013 | 0,018 | -0,009 | 0,01 |  |
| 8 | -0,015 | 0,021 | -0,01 | 0,011 |  |
| 9 | -0,015 | 0,021 | -0,011 | 0,012 |  |
| 10 | -0,016 | 0,018 | -0,012 | 0,012 |  |
| 11 | -0,018 | 0,014 | -0,012 | 0,013 |  |
| 12 | -0,019 | 0,013 | -0,013 | 0,013 |  |
| 13 | -0,02 | 0,009 | -0,012 | 0,013 |  |
| 14 | -0,02 | 0,011 | -0,013 | 0,014 |  |
| 15 | -0,022 | 0,008 | -0,014 | 0,014 |  |
| 16 | -0,023 | 0,006 | -0,014 | 0,015 |  |
| 17 | -0,024 | 0,007 | -0,014 | 0,015 |  |
| 18 | -0,025 | 0,006 | -0,015 | 0,016 |  |
| 19 | -0,026 | 0,004 | -0,015 | 0,016 |  |
| 20 | -0,027 | 0,003 | -0,016 | 0,016 |  |
| 21 | -0,028 | 0,003 | -0,016 | 0,016 |  |
| 22 | -0,029 | 0,003 | -0,016 | 0,016 |  |
| 23 | -0,03 | 0,004 | -0,017 | 0,017 |  |
| 24 | -0,03 | 0,002 | -0,017 | 0,017 |  |
| 25 | -0,032 | 0,002 | -0,018 | 0,017 |  |
| 26 | -0,032 | 0,003 | -0,018 | 0,018 |  |
| 27 | -0,033 | 0,002 | -0,018 | 0,018 |  |
| 28 | -0,034 | 0,002 | -0,019 | 0,019 |  |
| 29 | -0,034 | 0,002 | -0,02 | 0,019 |  |
| 30 | -0,034 | 0,002 | -0,019 | 0,018 |  |
| 31 | -0,035 | 0,002 | -0,019 | 0,02 |  |
| 32 | -0,035 | 0,002 | -0,019 | 0,019 |  |
| 33 | -0,036 | 0,002 | -0,02 | 0,019 |  |
| 34 | -0,036 | 0,003 | -0,02 | 0,02 |  |
| 35 | -0,036 | 0,002 | -0,02 | 0,02 |  |
| 36 | -0,036 | 0,002 | -0,02 | 0,02 |  |
| 37 | -0,036 | 0,003 | -0,021 | 0,02 |  |
| 38 | -0,036 | 0,003 | -0,02 | 0,02 |  |
| 39 | -0,036 | 0,003 | -0,021 | 0,02 |  |
| 40 | -0,036 | 0,003 | -0,021 | 0,021 |  |
| 41 | -0,035 | 0,003 | -0,02 | 0,02 |  |
| 42 | -0,036 | 0,002 | -0,02 | 0,02 |  |
| 43 | -0,035 | 0,003 | -0,02 | 0,02 |  |
| 44 | -0,035 | 0,003 | -0,02 | 0,019 |  |
| 45 | -0,035 | 0,003 | -0,02 | 0,02 |  |
| 46 | -0,035 | 0,003 | -0,02 | 0,02 |  |
| 47 | -0,034 | 0,003 | -0,02 | 0,019 |  |
| 48 | -0,032 | 0,004 | -0,019 | 0,019 |  |
| 49 | -0,032 | 0,004 | -0,019 | 0,019 |  |
| 50 | -0,032 | 0,004 | -0,019 | 0,019 |  |

These statistics correspond to the results presented in Figure 2. For all network densities, the differences were calculated as Group 2 – Group 1. Abbreviations: p-value, the 2-tailed p-value resulting from between-group comparison using permutation test with 10000 comparisons; CI-low and CI-high, the lower and higher bound of 95% confidence interval.

**Supplementary Table S2. Significant between-group differences in global efficiency evaluated at individual network densities.**

| CN Aβ- vs. AD Aβ+ | | | | |  |
| --- | --- | --- | --- | --- | --- |
|  |  |  |  |  |  |
| Delay 2 | | | | |  |
|  |  |  |  |  |  |
| Density (%) | Difference | p-value | CI-low | CI-high |  |
| 5 | -0,028 | 0,043 | -0,024 | 0,021 |  |
| 20 | -0,008 | 0,045 | -0,007 | 0,006 |  |
| 21 | -0,007 | 0,05 | -0,007 | 0,006 |  |
| 22 | -0,007 | 0,049 | -0,006 | 0,005 |  |
| 24 | -0,006 | 0,043 | -0,005 | 0,004 |  |
| 27 | -0,004 | 0,049 | -0,004 | 0,003 |  |
| Delay 3 | | | | |  |
|  |  |  |  |  |  |
| Density (%) | Difference | p-value | CI-low | CI-high |  |
| 5 | -0,038 | 0,007 | -0,024 | 0,022 |  |
| 6 | -0,032 | 0,013 | -0,022 | 0,02 |  |
| 7 | -0,031 | 0,012 | -0,022 | 0,019 |  |
| 8 | -0,027 | 0,015 | -0,019 | 0,016 |  |
| 9 | -0,026 | 0,012 | -0,018 | 0,015 |  |
| 10 | -0,022 | 0,01 | -0,016 | 0,013 |  |
| 11 | -0,022 | 0,007 | -0,014 | 0,012 |  |
| 12 | -0,02 | 0,011 | -0,013 | 0,011 |  |
| 13 | -0,018 | 0,009 | -0,012 | 0,01 |  |
| 14 | -0,017 | 0,006 | -0,011 | 0,009 |  |
| 15 | -0,015 | 0,007 | -0,01 | 0,008 |  |
| 16 | -0,014 | 0,007 | -0,009 | 0,008 |  |
| 17 | -0,013 | 0,005 | -0,009 | 0,007 |  |
| 18 | -0,012 | 0,007 | -0,008 | 0,007 |  |
| 19 | -0,011 | 0,009 | -0,008 | 0,006 |  |
| 20 | -0,01 | 0,008 | -0,006 | 0,006 |  |
| 21 | -0,009 | 0,009 | -0,006 | 0,005 |  |
| 22 | -0,008 | 0,008 | -0,006 | 0,005 |  |
| 23 | -0,008 | 0,008 | -0,005 | 0,004 |  |
| 24 | -0,007 | 0,007 | -0,005 | 0,004 |  |
| 25 | -0,007 | 0,007 | -0,004 | 0,004 |  |
| 26 | -0,006 | 0,006 | -0,004 | 0,003 |  |
| 27 | -0,006 | 0,008 | -0,004 | 0,003 |  |
| 28 | -0,005 | 0,008 | -0,003 | 0,003 |  |
| 29 | -0,005 | 0,006 | -0,003 | 0,003 |  |
| 30 | -0,004 | 0,015 | -0,003 | 0,002 |  |
| 31 | -0,004 | 0,008 | -0,003 | 0,002 |  |
| 32 | -0,003 | 0,011 | -0,002 | 0,002 |  |
| 33 | -0,003 | 0,012 | -0,002 | 0,002 |  |
| 34 | -0,003 | 0,012 | -0,002 | 0,001 |  |
| 35 | -0,002 | 0,013 | -0,002 | 0,001 |  |
| 36 | -0,002 | 0,017 | -0,001 | 0,001 |  |
| 37 | -0,001 | 0,024 | -0,001 | 0,001 |  |
| 38 | -0,001 | 0,04 | -0,001 | 0,001 |  |
| Delay 4 | | | | |  |
|  |  |  |  |  |  |
| Density (%) | Difference | p-value | CI-low | CI-high |  |
| 5 | -0,041 | 0,005 | -0,025 | 0,024 |  |
| 6 | -0,036 | 0,007 | -0,022 | 0,022 |  |
| 7 | -0,032 | 0,01 | -0,021 | 0,02 |  |
| 8 | -0,03 | 0,007 | -0,019 | 0,018 |  |
| 9 | -0,026 | 0,008 | -0,016 | 0,016 |  |
| 10 | -0,025 | 0,005 | -0,016 | 0,014 |  |
| 11 | -0,022 | 0,007 | -0,014 | 0,013 |  |
| 12 | -0,021 | 0,003 | -0,012 | 0,012 |  |
| 13 | -0,02 | 0,003 | -0,012 | 0,011 |  |
| 14 | -0,018 | 0,006 | -0,011 | 0,01 |  |
| 15 | -0,017 | 0,003 | -0,01 | 0,009 |  |
| 16 | -0,016 | 0,003 | -0,009 | 0,009 |  |
| 17 | -0,015 | 0,003 | -0,008 | 0,008 |  |
| 18 | -0,014 | 0,005 | -0,008 | 0,007 |  |
| 19 | -0,013 | 0,004 | -0,008 | 0,007 |  |
| 20 | -0,012 | 0,005 | -0,007 | 0,007 |  |
| 21 | -0,011 | 0,004 | -0,006 | 0,006 |  |
| 22 | -0,01 | 0,004 | -0,006 | 0,005 |  |
| 23 | -0,009 | 0,005 | -0,006 | 0,005 |  |
| 24 | -0,008 | 0,004 | -0,005 | 0,005 |  |
| 25 | -0,007 | 0,007 | -0,005 | 0,004 |  |
| 26 | -0,007 | 0,005 | -0,004 | 0,004 |  |
| 27 | -0,006 | 0,005 | -0,004 | 0,003 |  |
| 28 | -0,005 | 0,007 | -0,003 | 0,003 |  |
| 29 | -0,005 | 0,008 | -0,003 | 0,003 |  |
| 30 | -0,004 | 0,006 | -0,003 | 0,002 |  |
| 31 | -0,004 | 0,01 | -0,003 | 0,002 |  |
| 32 | -0,003 | 0,01 | -0,002 | 0,002 |  |
| 33 | -0,003 | 0,013 | -0,002 | 0,002 |  |
| 34 | -0,002 | 0,021 | -0,002 | 0,002 |  |
| 35 | -0,002 | 0,03 | -0,002 | 0,001 |  |
| 36 | -0,002 | 0,027 | -0,001 | 0,001 |  |
| 37 | -0,001 | 0,038 | -0,001 | 0,001 |  |
| CN Aβ- vs. CN Aβ+ | | | | |  |
|  |  |  |  |  |  |
| Delay 2 | | | | |  |
|  |  |  |  |  |  |
| Density (%) | Difference | p-value | CI-low | CI-high |  |
| 24 | -0,004 | 0,048 | -0,003 | 0,003 |  |
| 25 | -0,004 | 0,048 | -0,003 | 0,003 |  |
| 26 | -0,003 | 0,047 | -0,003 | 0,003 |  |
| 27 | -0,003 | 0,046 | -0,003 | 0,002 |  |
| 28 | -0,003 | 0,049 | -0,002 | 0,002 |  |
| 29 | -0,002 | 0,049 | -0,002 | 0,002 |  |
| 31 | -0,002 | 0,048 | -0,002 | 0,002 |  |
| Delay 3 | | | | |  |
|  |  |  |  |  |  |
| Density (%) | Difference | p-value | CI-low | CI-high |  |
| 8 | -0,014 | 0,042 | -0,012 | 0,012 |  |
| 9 | -0,014 | 0,032 | -0,011 | 0,01 |  |
| 10 | -0,013 | 0,033 | -0,01 | 0,01 |  |
| 11 | -0,012 | 0,023 | -0,009 | 0,008 |  |
| 12 | -0,011 | 0,02 | -0,008 | 0,008 |  |
| 13 | -0,011 | 0,014 | -0,007 | 0,007 |  |
| 14 | -0,01 | 0,013 | -0,007 | 0,007 |  |
| 15 | -0,009 | 0,012 | -0,006 | 0,006 |  |
| 16 | -0,009 | 0,015 | -0,006 | 0,006 |  |
| 17 | -0,008 | 0,012 | -0,006 | 0,005 |  |
| 18 | -0,007 | 0,018 | -0,005 | 0,005 |  |
| 19 | -0,007 | 0,015 | -0,005 | 0,005 |  |
| 20 | -0,006 | 0,015 | -0,004 | 0,004 |  |
| 21 | -0,006 | 0,011 | -0,004 | 0,004 |  |
| 22 | -0,005 | 0,02 | -0,004 | 0,004 |  |
| 23 | -0,005 | 0,022 | -0,004 | 0,003 |  |
| 24 | -0,005 | 0,019 | -0,003 | 0,003 |  |
| 25 | -0,004 | 0,016 | -0,003 | 0,003 |  |
| 26 | -0,004 | 0,031 | -0,003 | 0,003 |  |
| 27 | -0,003 | 0,026 | -0,003 | 0,002 |  |
| 28 | -0,003 | 0,03 | -0,002 | 0,002 |  |
| 29 | -0,003 | 0,025 | -0,002 | 0,002 |  |
| 31 | -0,002 | 0,028 | -0,002 | 0,002 |  |
| 32 | -0,002 | 0,035 | -0,002 | 0,001 |  |
| 33 | -0,002 | 0,04 | -0,001 | 0,001 |  |
| 34 | -0,001 | 0,031 | -0,001 | 0,001 |  |
| CN Aβ- vs MCI Aβ+ | | | | |  |
|  |  |  |  |  |  |
| Delay 5 | | | | |  |
|  |  |  |  |  |  |
| Density (%) | Difference | p-value | CI-low | CI-high |  |
| 5 | 0,02 | 0,044 | -0,017 | 0,016 |  |
| 7 | 0,016 | 0,046 | -0,014 | 0,013 |  |
| 14 | 0,008 | 0,045 | -0,007 | 0,007 |  |
| 15 | 0,008 | 0,04 | -0,006 | 0,006 |  |
| 16 | 0,007 | 0,045 | -0,006 | 0,006 |  |
| 17 | 0,007 | 0,043 | -0,006 | 0,005 |  |
| 18 | 0,006 | 0,043 | -0,005 | 0,005 |  |
| 19 | 0,006 | 0,045 | -0,005 | 0,005 |  |
| 20 | 0,005 | 0,044 | -0,004 | 0,004 |  |
| 21 | 0,005 | 0,039 | -0,004 | 0,004 |  |
| 22 | 0,005 | 0,034 | -0,004 | 0,004 |  |
| 24 | 0,004 | 0,037 | -0,003 | 0,003 |  |
| 25 | 0,004 | 0,04 | -0,003 | 0,003 |  |
| 26 | 0,003 | 0,045 | -0,003 | 0,003 |  |
| 27 | 0,003 | 0,037 | -0,002 | 0,002 |  |
| 28 | 0,002 | 0,049 | -0,002 | 0,002 |  |
| 40 | 0,001 | 0,04 | -0,001 | 0 |  |
| CN Aβ+ vs MCI Aβ+ | | | | |  |
|  |  |  |  |  |  |
| Delay 3 | | | | |  |
|  |  |  |  |  |  |
| Density (%) | Difference | p-value | CI-low | CI-high |  |
| 15 | 0,011 | 0,043 | -0,009 | 0,009 |  |
| 17 | 0,009 | 0,047 | -0,008 | 0,008 |  |
| 20 | 0,007 | 0,049 | -0,006 | 0,006 |  |
| 21 | 0,007 | 0,05 | -0,006 | 0,006 |  |
| 25 | 0,005 | 0,043 | -0,004 | 0,004 |  |
| 35 | 0,002 | 0,046 | -0,001 | 0,001 |  |
| 39 | 0,001 | 0,046 | -0,001 | 0,001 |  |
| Delay 4 | | | | |  |
|  |  |  |  |  |  |
| Density (%) | Difference | p-value | CI-low | CI-high |  |
| 8 | 0,02 | 0,049 | -0,017 | 0,017 |  |
| 10 | 0,016 | 0,048 | -0,013 | 0,014 |  |
| 11 | 0,014 | 0,048 | -0,012 | 0,012 |  |
| 12 | 0,013 | 0,037 | -0,011 | 0,01 |  |
| 13 | 0,012 | 0,043 | -0,01 | 0,01 |  |
| 14 | 0,011 | 0,041 | -0,009 | 0,009 |  |
| 15 | 0,011 | 0,036 | -0,008 | 0,008 |  |
| 16 | 0,009 | 0,04 | -0,008 | 0,007 |  |
| 17 | 0,009 | 0,05 | -0,007 | 0,007 |  |
| 38 | 0,001 | 0,043 | -0,001 | 0,001 |  |
| 43 | 0,001 | 0,028 | -0,001 | 0,001 |  |
| 48 | 0,001 | 0,014 | 0 | 0 |  |
| Delay 5 | | | | |  |
|  |  |  |  |  |  |
| Density (%) | Difference | p-value | CI-low | CI-high |  |
| 20 | 0,007 | 0,048 | -0,006 | 0,006 |  |
| 21 | 0,007 | 0,035 | -0,005 | 0,005 |  |
| 22 | 0,006 | 0,038 | -0,005 | 0,005 |  |
| 23 | 0,006 | 0,049 | -0,005 | 0,005 |  |
| 24 | 0,005 | 0,041 | -0,004 | 0,004 |  |
| 26 | 0,004 | 0,045 | -0,004 | 0,004 |  |
| 27 | 0,004 | 0,036 | -0,003 | 0,003 |  |
| 40 | 0,001 | 0,009 | -0,001 | 0,001 |  |
| MCI Aβ+ vs. AD Aβ+ | | | | |  |
|  |  |  |  |  |  |
| Delay 1 | | | | |  |
|  |  |  |  |  |  |
| Density (%) | Difference | p-value | CI-low | CI-high |  |
| 5 | -0,033 | 0,039 | -0,028 | 0,026 |  |
| 6 | -0,033 | 0,032 | -0,027 | 0,026 |  |
| 7 | -0,032 | 0,035 | -0,027 | 0,025 |  |
| 8 | -0,033 | 0,021 | -0,026 | 0,024 |  |
| 9 | -0,032 | 0,015 | -0,025 | 0,022 |  |
| 10 | -0,029 | 0,021 | -0,024 | 0,02 |  |
| 11 | -0,026 | 0,015 | -0,021 | 0,018 |  |
| 12 | -0,025 | 0,011 | -0,019 | 0,016 |  |
| 13 | -0,024 | 0,01 | -0,018 | 0,016 |  |
| 14 | -0,019 | 0,017 | -0,014 | 0,013 |  |
| 15 | -0,018 | 0,017 | -0,014 | 0,013 |  |
| 16 | -0,016 | 0,013 | -0,011 | 0,011 |  |
| 17 | -0,015 | 0,013 | -0,011 | 0,01 |  |
| 18 | -0,015 | 0,009 | -0,01 | 0,01 |  |
| 19 | -0,013 | 0,016 | -0,01 | 0,009 |  |
| 20 | -0,012 | 0,021 | -0,009 | 0,008 |  |
| 21 | -0,011 | 0,019 | -0,008 | 0,008 |  |
| 22 | -0,011 | 0,019 | -0,008 | 0,007 |  |
| 23 | -0,01 | 0,018 | -0,007 | 0,007 |  |
| 24 | -0,009 | 0,015 | -0,007 | 0,006 |  |
| 25 | -0,008 | 0,021 | -0,006 | 0,006 |  |
| 26 | -0,008 | 0,021 | -0,006 | 0,006 |  |
| 27 | -0,007 | 0,026 | -0,006 | 0,005 |  |
| 28 | -0,006 | 0,023 | -0,005 | 0,005 |  |
| 29 | -0,006 | 0,018 | -0,005 | 0,004 |  |
| 30 | -0,005 | 0,024 | -0,004 | 0,004 |  |
| 31 | -0,005 | 0,025 | -0,004 | 0,003 |  |
| 32 | -0,004 | 0,023 | -0,003 | 0,003 |  |
| 33 | -0,004 | 0,03 | -0,003 | 0,003 |  |
| 34 | -0,003 | 0,03 | -0,003 | 0,002 |  |
| 35 | -0,003 | 0,037 | -0,002 | 0,002 |  |
| 36 | -0,002 | 0,036 | -0,002 | 0,002 |  |
| 37 | -0,002 | 0,026 | -0,002 | 0,002 |  |
| 38 | -0,002 | 0,026 | -0,002 | 0,001 |  |
| 39 | -0,002 | 0,025 | -0,001 | 0,001 |  |
| 40 | -0,001 | 0,033 | -0,001 | 0,001 |  |
| 41 | -0,001 | 0,018 | -0,001 | 0,001 |  |
| 42 | -0,001 | 0,043 | -0,001 | 0,001 |  |
| 43 | -0,001 | 0,003 | -0,001 | 0,001 |  |
| 47 | -0,001 | 0,014 | 0 | 0 |  |
| 49 | -0,001 | 0,004 | 0 | 0 |  |
| 50 | 0 | 0,003 | 0 | 0 |  |
| Delay 3 | | | | |  |
|  |  |  |  |  |  |
| Density (%) | Difference | p-value | CI-low | CI-high |  |
| 5 | -0,04 | 0,027 | -0,031 | 0,03 |  |
| 6 | -0,034 | 0,038 | -0,029 | 0,026 |  |
| 7 | -0,033 | 0,042 | -0,028 | 0,026 |  |
| 8 | -0,029 | 0,044 | -0,025 | 0,023 |  |
| 9 | -0,027 | 0,045 | -0,024 | 0,022 |  |
| 10 | -0,024 | 0,03 | -0,02 | 0,019 |  |
| 11 | -0,023 | 0,032 | -0,019 | 0,017 |  |
| 12 | -0,02 | 0,043 | -0,018 | 0,016 |  |
| 13 | -0,019 | 0,038 | -0,016 | 0,015 |  |
| 14 | -0,018 | 0,033 | -0,015 | 0,013 |  |
| 15 | -0,016 | 0,032 | -0,014 | 0,012 |  |
| 16 | -0,015 | 0,031 | -0,013 | 0,011 |  |
| 17 | -0,014 | 0,028 | -0,012 | 0,011 |  |
| 18 | -0,013 | 0,029 | -0,01 | 0,01 |  |
| 19 | -0,012 | 0,04 | -0,01 | 0,009 |  |
| 20 | -0,011 | 0,026 | -0,009 | 0,008 |  |
| 21 | -0,01 | 0,042 | -0,009 | 0,008 |  |
| 22 | -0,009 | 0,036 | -0,007 | 0,007 |  |
| 23 | -0,008 | 0,037 | -0,007 | 0,007 |  |
| 24 | -0,008 | 0,034 | -0,006 | 0,006 |  |
| 25 | -0,008 | 0,024 | -0,006 | 0,006 |  |
| 26 | -0,007 | 0,03 | -0,006 | 0,005 |  |
| 27 | -0,006 | 0,036 | -0,005 | 0,005 |  |
| 28 | -0,006 | 0,033 | -0,005 | 0,004 |  |
| 29 | -0,005 | 0,022 | -0,004 | 0,004 |  |
| 30 | -0,004 | 0,04 | -0,004 | 0,003 |  |
| 31 | -0,004 | 0,028 | -0,004 | 0,003 |  |
| 32 | -0,004 | 0,04 | -0,003 | 0,003 |  |
| 33 | -0,003 | 0,03 | -0,003 | 0,002 |  |
| 34 | -0,003 | 0,029 | -0,003 | 0,002 |  |
| 35 | -0,003 | 0,014 | -0,002 | 0,002 |  |
| 36 | -0,002 | 0,021 | -0,002 | 0,002 |  |
| 37 | -0,002 | 0,016 | -0,001 | 0,001 |  |
| 38 | -0,002 | 0,036 | -0,001 | 0,001 |  |
| 39 | -0,001 | 0,038 | -0,001 | 0,001 |  |
| 40 | -0,001 | 0,016 | -0,001 | 0,001 |  |
| 41 | -0,001 | 0,014 | -0,001 | 0,001 |  |
| 43 | -0,001 | 0,013 | -0,001 | 0,001 |  |
| 45 | 0 | 0,016 | 0 | 0 |  |
| 46 | 0 | 0,013 | 0 | 0 |  |
| 47 | 0 | 0,044 | 0 | 0 |  |
| 50 | 0 | 0,006 | 0 | 0 |  |
| Delay 4 | | | | |  |
|  |  |  |  |  |  |
| Density (%) | Difference | p-value | CI-low | CI-high |  |
| 5 | -0,049 | 0,003 | -0,029 | 0,028 |  |
| 6 | -0,043 | 0,003 | -0,026 | 0,025 |  |
| 7 | -0,041 | 0,002 | -0,023 | 0,023 |  |
| 8 | -0,037 | 0,002 | -0,022 | 0,02 |  |
| 9 | -0,034 | 0,002 | -0,019 | 0,018 |  |
| 10 | -0,032 | 0,001 | -0,018 | 0,017 |  |
| 11 | -0,028 | 0,001 | -0,016 | 0,015 |  |
| 12 | -0,027 | 0,001 | -0,015 | 0,014 |  |
| 13 | -0,025 | 0,001 | -0,014 | 0,013 |  |
| 14 | -0,022 | 0,002 | -0,013 | 0,012 |  |
| 15 | -0,022 | 0,001 | -0,012 | 0,011 |  |
| 16 | -0,02 | 0,002 | -0,011 | 0,011 |  |
| 17 | -0,018 | 0,002 | -0,01 | 0,01 |  |
| 18 | -0,017 | 0,002 | -0,009 | 0,009 |  |
| 19 | -0,016 | 0,002 | -0,009 | 0,008 |  |
| 20 | -0,014 | 0,003 | -0,008 | 0,008 |  |
| 21 | -0,013 | 0,003 | -0,008 | 0,007 |  |
| 22 | -0,012 | 0,003 | -0,007 | 0,007 |  |
| 23 | -0,011 | 0,002 | -0,006 | 0,006 |  |
| 24 | -0,01 | 0,003 | -0,006 | 0,006 |  |
| 25 | -0,009 | 0,003 | -0,006 | 0,005 |  |
| 26 | -0,009 | 0,003 | -0,005 | 0,005 |  |
| 27 | -0,008 | 0,003 | -0,005 | 0,004 |  |
| 28 | -0,007 | 0,004 | -0,004 | 0,004 |  |
| 29 | -0,006 | 0,005 | -0,004 | 0,004 |  |
| 30 | -0,005 | 0,004 | -0,004 | 0,003 |  |
| 31 | -0,005 | 0,005 | -0,003 | 0,003 |  |
| 32 | -0,004 | 0,005 | -0,003 | 0,003 |  |
| 33 | -0,004 | 0,004 | -0,002 | 0,002 |  |
| 34 | -0,003 | 0,003 | -0,002 | 0,002 |  |
| 35 | -0,003 | 0,007 | -0,002 | 0,002 |  |
| 36 | -0,002 | 0,006 | -0,002 | 0,001 |  |
| 37 | -0,002 | 0,007 | -0,001 | 0,001 |  |
| 38 | -0,002 | 0,002 | -0,001 | 0,001 |  |
| 39 | -0,001 | 0,003 | -0,001 | 0,001 |  |
| 40 | -0,001 | 0,002 | -0,001 | 0,001 |  |
| 41 | -0,001 | 0,003 | -0,001 | 0,001 |  |
| 42 | -0,001 | 0,013 | -0,001 | 0 |  |
| 43 | -0,001 | 0,001 | 0 | 0 |  |
| 44 | 0 | 0,026 | 0 | 0 |  |
| 47 | 0 | 0,024 | 0 | 0 |  |
| 50 | 0 | 0,007 | 0 | 0 |  |
| Delay 5 | | | | |  |
|  |  |  |  |  |  |
| Density (%) | Difference | p-value | CI-low | CI-high |  |
| 5 | -0,041 | 0,003 | -0,024 | 0,023 |  |
| 6 | -0,037 | 0,004 | -0,023 | 0,021 |  |
| 7 | -0,033 | 0,004 | -0,02 | 0,019 |  |
| 8 | -0,028 | 0,008 | -0,019 | 0,018 |  |
| 9 | -0,025 | 0,01 | -0,018 | 0,016 |  |
| 10 | -0,023 | 0,01 | -0,017 | 0,015 |  |
| 11 | -0,02 | 0,008 | -0,014 | 0,013 |  |
| 12 | -0,02 | 0,005 | -0,013 | 0,012 |  |
| 13 | -0,018 | 0,005 | -0,011 | 0,011 |  |
| 14 | -0,016 | 0,006 | -0,01 | 0,01 |  |
| 15 | -0,015 | 0,006 | -0,01 | 0,01 |  |
| 16 | -0,014 | 0,006 | -0,009 | 0,009 |  |
| 17 | -0,013 | 0,007 | -0,009 | 0,008 |  |
| 18 | -0,012 | 0,008 | -0,008 | 0,008 |  |
| 19 | -0,012 | 0,005 | -0,008 | 0,007 |  |
| 20 | -0,011 | 0,006 | -0,007 | 0,007 |  |
| 21 | -0,01 | 0,005 | -0,007 | 0,006 |  |
| 22 | -0,01 | 0,005 | -0,006 | 0,006 |  |
| 23 | -0,009 | 0,006 | -0,006 | 0,006 |  |
| 24 | -0,008 | 0,008 | -0,005 | 0,005 |  |
| 25 | -0,008 | 0,005 | -0,005 | 0,005 |  |
| 26 | -0,007 | 0,011 | -0,005 | 0,004 |  |
| 27 | -0,006 | 0,005 | -0,004 | 0,004 |  |
| 28 | -0,005 | 0,009 | -0,004 | 0,004 |  |
| 29 | -0,005 | 0,014 | -0,003 | 0,003 |  |
| 30 | -0,004 | 0,014 | -0,003 | 0,003 |  |
| 31 | -0,003 | 0,021 | -0,003 | 0,002 |  |
| 32 | -0,003 | 0,014 | -0,002 | 0,002 |  |
| 33 | -0,003 | 0,014 | -0,002 | 0,002 |  |
| 34 | -0,002 | 0,021 | -0,002 | 0,002 |  |
| 35 | -0,002 | 0,016 | -0,002 | 0,001 |  |
| 36 | -0,002 | 0,014 | -0,001 | 0,001 |  |
| 37 | -0,001 | 0,049 | -0,001 | 0,001 |  |
| 38 | -0,001 | 0,031 | -0,001 | 0,001 |  |
| 42 | -0,001 | 0,005 | 0 | 0 |  |
| 43 | 0 | 0,023 | 0 | 0 |  |
| 50 | 0 | 0,018 | 0 | 0 |  |

These statistics correspond to the results presented in Figure 2. For all network densities, the differences were calculated as Group 2 – Group 1. Abbreviations: p-value, the 2-tailed p-value resulting from between-group comparison using permutation test with 10000 comparisons; CI-low and CI-high, the lower and higher bound of 95% confidence interval.

**Supplementary Table S3. Variance Inflation Factors (VIF) between network measures as different temporal delays and age, sex, education and cognitive status.**

|  | Clustering coefficient | Global  efficiency | Age | Sex | Education | Cognitive  status |
| --- | --- | --- | --- | --- | --- | --- |
| Delay 1 | 1.81 | 1.89 | 1.25 | 1.14 | 1.36 | 1.10 |
| Delay 2 | 3.40 | 3.64 | 1.21 | 1.22 | 1.31 | 1.09 |
| Delay 3 | 2.86 | 2.96 | 1.25 | 1.21 | 1.31 | 1.09 |
| Delay 4 | 3.96 | 3.89 | 1.23 | 1.18 | 1.28 | 1.12 |
| Delay 5 | 2.38 | 2.31 | 1.23 | 1.15 | 1.25 | 1.13 |
